# Dynamic financial tail risk networks: A backtesting-based conditional expected shortfall approach

**DOI:** 10.1371/journal.pone.0351966

**Published:** 2026-06-24

**Authors:** Donghao Zhang, Xiaodong Yan, Feng Shen

**Affiliations:** 1 Chinese Financial Research Institute, Southwestern University of Finance and Economics, Chengdu, PR China; 2 School of Finance, Southwestern University of Finance and Economics, Chengdu, PR China; 3 Engineering Research Center of Intelligent Finance, Southwestern University of Finance and Economics, Chengdu, PR China; Sapienza University of Rome, ITALY

## Abstract

This paper develops a Factor-Copula methodology for constructing high-dimensional dynamic tail risk networks based on the conditional expected shortfall (CoES) in order to overcome the limitations of traditional quantile regression and copula models. We backtest the CoES using cumulative joint violations and conditional coverage tests. The proposed factor‑copula‑CoES model reduces the rejection rates in 10‑th order conditional backtests by 56.37%, 47.72%, and 1.96% relative to the static‑copula‑CoES, time‑varying‑copula‑CoES, and factor‑copula‑CoVaR models, respectively, with all reductions being statistically significant. The dynamic analysis of the tail risk network of Chinese listed financial institutions indicates that the network topology characteristics align with real market risk events. Finally, the quantitative analyses show different effects of institutional and market factors on network-measured risk spillovers and contagion among Chinese financial institutions.

## 1. Introduction

In 2008, the collapse of the U.S. subprime mortgage market triggered a global financial crisis, resulting in severe market fluctuations that undermined financial system stability and have since drawn widespread regulatory and academic attention to systemic risk. Since the early 1990s, regulators and institutions have relied on Value-at-Risk (VaR) to monitor tail risk and estimate maximum potential losses at a given confidence level. However, VaR fails to fully capture expected tail losses, lacks convexity and sub-additivity, and is neither coherent nor resistant to regulatory arbitrage [1]. Research has demonstrated that Expected Shortfall (ES) provides a superior measure by coherently assessing losses beyond a confidence threshold [2] and exhibiting enhanced tail sensitivity [3]. Because of the ES’s mathematical and financial robustness, the Basel Committee on Banking Supervision (BCBS) adopted ES in 2016 to replace VaR for capital adequacy calculations. However, both VaR and ES measure only individual institution risk, ignoring interconnections between institutions. To address this limitation, Adrian and Brunnermeier [4] proposed the Conditional Value-at-Risk (CoVaR) and Conditional Expected Shortfall (CoES) to measure tail risk spillover relationships between financial institutions. ${CoVaR}^{i|j}$ is defined as the VaR of institution i when institution j is in distress, while ${CoES}^{i|j}$ defines the ES of institution i under the same conditions. These metrics quantify the magnitude of risk spillover between financial institutions i and j, enabling the construction of tail risk spillover networks.

Network analysis is widely used in systemic financial risk research. However, conventional financial network construction requires detailed inter-institutional exposure data [5,6], which is rarely publicly available. Consequently, studies increasingly build tail-risk networks directly based on ${CoVaR}^{i|j}$ and ${CoES}^{i|j}$, with research by Hautsch et al. [7] and Härdle et al. [8] based on the CoVaR concept, and work by Chen et al. [9] and Bonaccolto et al. [10] based on the CoES. However, a common limitation in these studies is the reliance on quantile regression models, which often fail to model nonlinear and dynamic dependencies. Girardi and Ergün [11] employed the DCC-GARCH model for dynamic CoVaR estimation; however, its binary dependency structure assumptions have limited its applicability. Copula models address this by enabling more flexible dependency structures [12,13], yet both copula models and DCC-GARCH framework suffer from the curse of dimensionality in high-dimensional settings. Oh and Patton [14] introduced a Factor Copula model based on the GAS algorithm to aptly capture the high-dimensional dynamic dependencies between institutions. Recent advances in factor copula models span methodological innovation, structural change detection, and portfolio risk optimization. Opschoor et al. [15] introduced closed-form multi-factor copulas with observation-driven dynamic factor loadings, enabling scalable and flexible high‑dimensional dependence modeling. Chang et al. [16] developed a change‑point detection framework for dynamic factor copulas that identifies both smooth and abrupt structural breaks in dependence. Chen et al. [17] applied a dynamic factor copula‑based mean‑ES model to optimize portfolio risk in China’s real industry, showing the approach effectively captures time‑varying dependence in high‑dimensions. However, few studies have applied factor copulas specifically to the construction of financial risk networks. To fill this gap, we propose a Factor Copula–based framework for constructing high‑dimensional dynamic tail‑risk networks.

Backtesting is a cornerstone of risk model validation. Although robust frameworks exist for individual risk measures like VaR and ES [18,19], comparable validation frameworks for CoVaR and CoES remain underdeveloped. Girardi and Ergün [11] and Karimalis and Nomikos [13] applied CoVaR backtesting to validate the DCC-GARCH-CoVaR model and the Copula-CoVaR model. However, CoES backtesting is scarcely explored. To bridge this gap, we develop a cumulative joint violation process for backtesting CoES. This process integrates the cumulative violation process of Du and Escanciano [1] for backtesting ES and the joint violation process of Banulescu-Radu et al. [20] for backtesting CoVaR. Thus, by backtesting each edge in a CoVaR/CoES-defined tail-risk network, we ensure the reliability of the network as a whole.

We empirically test our methodology using weekly returns of 55 A-share listed financial institutions in China from January 2011 to December 2022. The sample covers three systemic risk events in China: (1) the 2013 interbank liquidity crunch, (2) the 2015–2016 stock market crash, and (3) the COVID-19 pandemic period (2020–2022). We compare tail risk networks built with three approaches: (i) high-dimensional dynamic Factor Copula, (ii) bivariate static Copula, and (iii) bivariate dynamic Copula. Results show that networks constructed with the Factor Copula consistently yield lower rejection rates in conditional coverage tests than alternatives, and CoES-based networks achieve lower rejection rates than CoVaR-based ones. Consequently, we employ the Factor-Copula-CoES model to analyze China’s financial systemic risk evolution across crisis periods. Dynamic network metrics (e.g., centrality, density) correlate strongly with actual market turbulence and exhibit clear risk clustering during crises. Network visualizations reveal risk clusters within and across financial sectors over time, indicating a strong sectoral clustering effect among highly correlated, high-risk institutions.

Finally, we investigate the factors influencing systemic risk at both institutional and market levels, measuring systemic risk through tail risk network characteristics. Compared to traditional systemic risk measures based on ${CoVaR}^{s|i}$ or ${CoES}^{s|i}$ (where s denotes overall systemic risk), our network-based approach offers two distinct advantages. First, conventional ${CoVaR}^{s|i}$-based studies typically approximate systemic returns using aggregate institutional returns, leading to potential information loss [14]. In contrast, our node centrality measures in tail risk networks preserve the complete risk spillover relationships among all institutions. Second, while ${CoVaR}^{s|i}$-based measures only capture unidirectional risk spillovers from institutions to the system, our centrality metrics (through out-degree and in-degree) capture both outgoing and incoming risks, thus providing a more comprehensive perspective on systemic risk factors. To demonstrate the utility of our network-based approach, we employ it to examine key determinants of systemic risk at both institutional and market levels. Existing literature reports mixed findings on the link between institutional size and systemic risk. Some studies report a positive correlation due to moral hazard [21], while others suggest larger institutions may pose lower systemic risk due to conflict of interest effects [22]. Smaller institutions, conversely, may exhibit greater risk vulnerability [23]. Additional financial indicators like liquidity [24,25] and leverage [26] also influence systemic risk levels. Furthermore, following Adrian and Brunnermeier [4], we incorporate the spread of China’s local government debt as a market-wide risk factor.

Compared with previous studies, this research contributes three key innovations: First, we propose a Factor-Copula-CoES-based method for constructing high-dimensional dynamic tail-risk networks. This approach simultaneously addresses two limitations: (1) the inability of quantile regression models to capture dynamic nonlinear dependencies, and (2) the curse of dimensionality in conventional high-dimensional Copula models. Second, we introduce a comprehensive backtesting framework for CoVaR and CoES to ensure the validity of both the risk measures and the constructed network. Unlike the indirect CoVaR and CoES estimations in traditional quantile regression, the Factor Copula model enables direct estimation, which in turn lays the foundation for our backtesting framework. Specifically, we combine the cumulative violation process for ES backtesting with the joint violation process for CoVaR backtesting to construct a novel joint cumulative violation process for CoES backtesting. Third, using tail-risk network characteristics as systemic risk proxies, we identify the key factors affecting systemic risk in China’s financial system. We then separately examine their influences on risk spillover and risk contagion. Our empirical results reveal that institutional-level and market-level factors have distinct impacts on risk spillover and contagion across financial institutions.

The remainder of this paper is organized as follows. Section 2 introduces the research methods, including the definitions for the CoVaR and CoES tail risk measures, the dynamic high-dimensional Factor Copula model, the backtesting framework, and the construction of the tail risk networks. Section 3 details the data and the backtesting results, and presents the constructed tail risk network and the dynamic characteristics. Section 4 explores the factors affecting systemic risk in China’s financial system by measuring the systemic risk by network characteristics. Finally, Section 5 concludes the paper and discusses the implications. Appendix contains the mathematical proofs and Monte Carlo simulations of our proposed CoES backtesting framework.

## 2. Methodology

### 2.1. CoVaR and CoES definitions

Let the random variable $R_{i,t}$ denote the return of institution i at time t (i=1,…,N;t=1,…,T). The Value-at-Risk (VaR) of institution i at time t at the given confidence level α ∈ (0, 1) is denoted by $\overset{i}{\underset{\alpha ,t}{VaR}}$ and is defined as the α-quantile of the $R_{i,t}$ distribution:


$$\begin{array}{c} \begin{array}{c} Pr\left(R_{i,t}\leq \overset{i}{\underset{\alpha ,t}{VaR}}\right)=\alpha \end{array} \end{array}$$ (1)

Let $F_{i,t}$ denote the cumulative distribution function (CDF) of $R_{i,t}$. Its inverse, the quantile function, is defined as $\overset{-\text{1}}{\underset{i,t}{F}}(\alpha )=inf\{r_{i,t}\in R:F_{i,t}(r_{i,t}\geq \alpha )\}$. Thus, Equation (1) can be equivalently expressed as:


$$\begin{array}{c} \overset{i}{\underset{\alpha ,t}{VaR}}=\overset{-\text{1}}{\underset{i,t}{F}}(\alpha ) \end{array}$$ (2)

Extending the concept to the bivariate random vector $\left[R_{i,t},R_{j,t}\right]$, the Conditional Value-at-Risk CoVaR of institution i given that institution j is in distress, denoted $\overset{i|j}{\underset{\beta |\alpha ,t}{CoVaR}}$, is originally defined by Adrian and Brunnermeier [4] in a linear quantile regression framework as:


$$\begin{array}{c} \begin{array}{c} Pr\left(R_{i,t}\leq \overset{i|j}{\underset{\beta |\alpha ,t}{CoVaR}}\;|\;R_{j,t}=\overset{j}{\underset{\alpha ,t}{VaR}}\right)=\beta \end{array} \end{array}$$ (3)

The original definition in equation (3) uses a strict conditioning event $R_{j,t}=\overset{j}{\underset{\alpha ,t}{VaR}}$, which is not conducive to backtesting. Following Girardi and Ergün [11], we adopt a revised version that broadens the conditioning event to $R_{j,t}\leq \overset{j}{\underset{\alpha ,t}{VaR}}$, facilitating estimation and backtesting within models like DCC-GARCH and copulas. Thus, the CoVaR definition used in this study is:


$$\begin{array}{c} \begin{array}{c} Pr\left(R_{i,t}\leq \overset{i|j}{\underset{\beta |\alpha ,t}{CoVaR}}\;|\;R_{j,t}\leq \overset{j}{\underset{\alpha ,t}{VaR}}\right)=\beta \end{array} \end{array}$$ (4)

Building on formula (4), the Conditional Expected Shortfall CoES is defined as the expected shortfall of institution i at level β conditional on institution j being in distress:


$$\begin{array}{c} \begin{array}{c} \overset{i|j}{\underset{\beta |\alpha ,t}{CoES}}=\;E\left(R_{i,t\;}|{\;R}_{i,t}\leq \overset{i|j}{\underset{\beta |\alpha ,t}{CoVaR}},R_{j,t}\leq \overset{j}{\underset{\alpha ,t}{VaR}}\right) \end{array} \end{array}$$ (5)

Compared to CoVaR, which only provides a quantile threshold, CoES captures the average of losses beyond that threshold. Thus, CoES offers a more comprehensive measure of the expected tail losses of institution i conditional on the distress of institution j.

### 2.2. Factor copula CoVaR and CoES methodology

This section outlines the estimation of ${CoVaR}^{i|j}$ and ${CoES}^{i|j}$ using a high-dimensional dynamic factor copula model. Although estimating ${CoVaR}^{i|j}$ and ${CoES}^{i|j}$ for a single pair (i, j) requires only a bivariate dependency and can be done with a traditional bivariate copula, constructing a tail-risk network demands consistent estimation of these measures for all institution pairs (i, j). A factor copula model, by contrast, estimates the joint distribution of the entire return vector ${𝐑}_{𝐭}=\;[R_{\text{1},t},R_{\text{2},t},\ldots ,R_{N,t}]$. This provides a coherent framework for extracting all pairwise dependencies from a single high-dimensional model, ensuring consistency across the network—an assurance that separate bivariate copula estimations cannot provide.

Let ${𝐑}_{𝐭}$ be an N-dimensional vector of returns at time t. Denote its joint distribution function by ${𝐅}_{𝐭}$, the marginal distribution of $R_{i,t}$ by $F_{i,t}$, and the copula function linking them by ${𝐂}_{𝐭}$. According to Sklar’s theorem [27], the joint distribution function ${𝐅}_{𝐭}$ can be decomposed into its marginal distributions $F_{i,t}$ and the Copula function ${𝐂}_{𝐭}$:


$$\begin{array}{c} \begin{array}{c} \left[R_{\text{1},\text{t}},R_{\text{2},t},\ldots ,R_{N,t}\right]\equiv {𝐑}_{𝐭}\sim {𝐅}_{𝐭}={𝐂}_{𝐭}\left(F_{\text{1},t},F_{\text{2},t},\ldots ,F_{N,t}\right) \end{array} \end{array}$$ (6)

Following Sklar’s theorem, we adopt a two-step estimation procedure: first model the marginal distributions $F_{i,t}$, then model the copula ${𝐂}_{𝐭}$. For the return $R_{i,t}$, it is conventionally assumed that the marginal distribution of the individual returns $R_{i,t}$ follows the AR(1)-GARCH(1,1)-Skewed-t model:


$$\begin{array}{c} \begin{array}{c} R_{i,t}=\mu_{i}+\phi_{i}R_{i,t-\text{1}}+e_{i,t} \end{array} \end{array}$$ (7)

$$\begin{array}{c} e_{i,t}=\sigma_{i,t}\eta_{i,t} \end{array}$$

$$\begin{array}{c} \begin{array}{c} \overset{\text{2}}{\underset{i,t}{\sigma }}=\omega_{i}+\beta_{i}\overset{\text{2}}{\underset{i,t-\text{1}}{\sigma }}+\alpha_{i}\overset{\text{2}}{\underset{i,t-\text{1}}{e}} \end{array} \end{array}$$ (8)

$$\begin{array}{c} \begin{array}{c} \;\eta_{i,t}\equiv \frac{e_{i,t}}{\sigma_{i,t}}\sim Skewed\;t\left(v_{i},\psi_{i}\right) \end{array} \end{array}$$ (9)

and equations (7)–(9) represent the mean equation, variance equation, and error distribution assumption of the GARCH model, respectively. Equation (7) is an AR(1) process that describes the mean dynamics of the return series $R_{i,t}$, where $\mu_{i}$ is the intercept, $\phi_{i}$ is the first-order autoregressive coefficient ($|\phi_{i}|<\text{1}$), and $e_{i,t}$ is the residual at time t. The conditional distribution of the residual is given by $e_{i,t}=\sigma_{i,t}\eta_{i,t}$, where $\sigma_{i,t}$ is the conditional standard deviation (determined by equation 8) and $\eta_{i,t}$ is the standardized residual, which follows a standard skewed t distribution (specified by equation 9). Equation (8) is a GARCH(1,1) process that describes the dynamics of the conditional variance, where $\overset{\text{2}}{\underset{i,t}{\sigma }}$ is the conditional variance (volatility) at time t, $\omega_{i}$ is the constant term ($\omega_{i}>\text{0}$), $\alpha_{i}$ is the ARCH term coefficient ($\alpha_{i}\geq \text{0}$), and $\beta_{i}$ is the GARCH term coefficient ($\beta_{i}\geq \text{0}$), $\alpha_{i}+\beta_{i}<\text{1}$. Equation (9) specifies that the standardized residual $\eta_{i,t}$ follows a standardized skewed t distribution with degrees of freedom $v_{i}$ and skewness parameter $\psi_{i}$.

Next, utilizing the high-dimensional Dynamic Factor Copula model, the Copula function ${𝐂}_{𝐭}$ is modeled following Oh and Patton [14], the joint standardized errors $\eta_{t}$≡$\left[\eta_{\text{1},t},\ldots ,\eta_{N,t}\right]$ and the latent variable ${𝐗}_{𝐭}$≡$\left[X_{\text{1},t},\ldots ,X_{N,t}\right]$ share the same copula function, with the links between $X_{it}$ being driven by the common factor $Z_{t}$:


$$\begin{array}{c} X_{i,t}=\lambda_{i,t}\left(\gamma_{\lambda }\right)Z_{t}+\;\varepsilon_{i,t}\;, \end{array}$$

$$\begin{array}{c} \begin{array}{c} \text{ where }Z_{t}\;\sim \;Skewed\;t\left(v_{Z},\psi_{Z}\right),\;\varepsilon_{i,t}\;\sim \;i.i.d.t\left(v_{\varepsilon }\right),\;Z_{t}⊥\varepsilon_{i,t}\forall \;i \end{array} \end{array}$$ (10)

where the common factor $Z_{t}$ follows a skewed t-distribution with $v_{Z}$ degrees of freedom and $\psi_{Z}$ skewness. The idiosyncratic factor $\varepsilon_{i,t}$ is distributed as an i.i.d. standard t-distribution with $v_{\varepsilon }$ degrees of freedom. For any given i, $Z_{t}⊥\varepsilon_{it}$. The factor loading, $\lambda_{i,t}$, is a potentially time-varying weight on the common factor, and we denote the parameters of the factor loading $\lambda_{i,t}$ as $\gamma_{\lambda }$. We employ the Generalized Autoregressive Score (GAS) model to allow for time variation in the factor loadings in the factor copula implied by equation (10).

As an ARCH-type model, the GAS framework offers a distinct advantage because it circumvents the need to integrate out the innovation terms driving latent processes—a feature particularly valuable in high-dimensional applications. Moreover, compared to alternative ARCH-type models, the GAS approach is better suited for contexts where parameters lack immediate intuitive interpretation, such as in high-dimensional copula models [14]. In the general GAS framework, consider a copula with a time-varying parameter vector $\delta_{t}$, which is driven by a vector of fixed parameters γ, we have:


$$\begin{array}{c} Let\;{𝐔}_{t}|\mathrm{\backslash pmb}{ℱ}_{t-\text{1}}\sim \;𝐂(\delta_{t}(\gamma )) \end{array}$$

$$\begin{array}{c} then\;\delta_{t}=\omega +B\delta_{t-\text{1}}+As_{t-\text{1}} \end{array}$$

$$\begin{array}{c} where\;s_{t-\text{1}}\;=\;S_{t-\text{1}}\nabla_{t-\text{1}} \end{array}$$

$$\begin{array}{c} \begin{array}{c} \;\nabla_{t-\text{1}}=\frac{\partial logc\left(u_{t-\text{1}};\delta_{t-\text{1}}\right)}{\partial \delta_{t-\text{1}}} \end{array} \end{array}$$ (11)

where $\mathrm{\backslash pmb}F_{t}$ denote the information set containing all available information up to and including time t,

${𝐔}_{t}=[U_{\text{1},t},\ldots ,U_{N,t}]$, $U_{i,t}$ is the conditional probability integral transforms of $R_{i,t}$, 𝐂 is the copula function. The relation ${𝐔}_{t}|\mathrm{\backslash pmb}F_{t-\text{1}}\sim \;𝐂(\delta_{t}(\gamma ))$ implys that the conditional copula of ${𝐑}_{t}|\mathrm{\backslash pmb}F_{t-\text{1}}$ is equal to the conditional distribution of ${𝐔}_{t}|\mathrm{\backslash pmb}F_{t-\text{1}}$. ω, B, and A are the parameters to be estimated, $S_{t}$ is a scaling matrix (e.g., the inverse Hessian or its square root).

To maintain model parsimony, we restrict the coefficient matrices B and A in formula (11) to be diagonal with a common scalar parameter along their respective diagonals. Furthermore, to circumvent the estimation of an N × N scaling matrix, we set $S_{t}=I$. Under these specifications, the model reduces to the following log form:


$$\begin{array}{c} \begin{array}{c} log\;\lambda_{i,t}=\omega_{\lambda i}+\beta_{\lambda }\;log\;\lambda_{i,t-\text{1}}+\alpha_{\lambda }s_{i,t-\text{1}},i=\text{1},\text{2},\ldots ,N \end{array} \end{array}$$ (12)

where $s_{i,t}\equiv \partial log\;c(u_{t};\lambda_{t},v_{\varepsilon },\psi_{z},v_{Z})/\partial log\;\lambda_{i,t}$ and $\lambda_{t}\equiv [\lambda_{\text{1},t},\ldots ,\lambda_{N,t}]$. In this high-dimensional dynamic Factor Copula model, there are a total of N + 5 parameters: $\beta_{\lambda },\alpha_{\lambda },v_{z},\psi_{z},v_{\varepsilon },{\;\omega }_{\lambda i}\;,i=\text{1},\text{2},\ldots ,N$.

While the Copula function c and its likelihood are not known in a closed form, this can be approximated using numerical integration. The approximated likelihood is then maximized via maximum likelihood estimation (MLE) to obtain estimates of all model parameters. When the high-dimensional dynamic copula model parameter estimations are completed, data ${𝐗}_{𝐭}$ can be easily simulated for the implicit dynamic copula function ${𝐂}_{𝐭}$ using Equation (10). Then an inverse probability integral transformation can be performed on $X_{i,t}$, by inverting the transformations from Equations (9), (8), and (7), a set of simulations for ${𝐑}_{𝐭}$ are obtained. Through extensive simulations, it is then possible to acquire the simulated joint distribution for ${𝐑}_{𝐭}$, which allows for the estimation to be made for the ${CoVaR}^{i|j}$ and ${CoES}^{i|j}$ measures based on the simulated joint distributions of $\left[R_{i,t},R_{j,t}\right]$.

### 2.3. CoVaR and CoES backtesting

This section explains the backtesting CoVaR and CoES frameworks. First, following the approach by Banulescu et al. (2020), the joint violation process for backtesting CoVaR was constructed:


$$\begin{array}{c} \begin{array}{c} h_{t}(\alpha ,\beta ,\theta )=\text{1}\left(\left(R_{i,t}\leq \overset{i|j}{\underset{\beta |\alpha ,t}{CoVaR}}\right)\cap \;\left(R_{j,t}\leq \overset{j}{\underset{\alpha ,t}{VaR}}\right)\right) \end{array} \end{array}$$ (13)

where θ represents all unknown parameters within the CoVaR estimation model, and 1(·) is an indicator function. If the CoVaR model is appropriate, then for any β∈[0,1], the series $\overset{\infty }{\underset{t=\text{1}}{\left\{h_{t}(\alpha ,\beta ,\theta )-\alpha \beta \right\}}}$ should be a martingale difference sequence (MDS).

Noting that $\overset{i|j}{\underset{\beta |\alpha ,t}{CoES}}=\frac{\text{1}}{\beta }\int_{\text{0}}^{\beta }\overset{i|j}{\underset{\beta |\alpha ,t}{CoVaR}}dz$, to verify the correct specification for the CoES model, this study considers the integral of $h_{t}$ and constructs a cumulative joint violation process $H_{t}$ as:


$$\begin{array}{c} \begin{array}{c} H_{t}(\alpha ,\beta ,\theta )=\frac{\text{1}}{\beta }\int_{\text{0}}^{\beta }h_{t}(\alpha ,\kappa ,\theta )d\kappa \end{array} \end{array}$$ (14)

Given Fubini’s theorem, we have:


$$\begin{array}{c} \begin{array}{c} E\left[H_{t}(\alpha ,\beta ,\theta )\right]=E\left[\;\frac{\text{1}}{\beta }\int_{\text{0}}^{\beta }h_{t}(\alpha ,\kappa ,\theta )d\kappa \right]=\frac{\text{1}}{\beta }\int_{\text{0}}^{\beta }E\left[h_{t}(\alpha ,\kappa ,\theta )\right]d\kappa \end{array} \end{array}$$ (15)

$$\begin{array}{c} \begin{array}{c} \;=\frac{\text{1}}{\beta }\int_{\text{0}}^{\beta }\alpha \kappa d\kappa =\frac{\text{1}}{\beta }\left(\alpha *\frac{\beta^{\text{2}}}{\text{2}}\right)=\frac{\alpha \beta }{\text{2}} \end{array} \end{array}$$

it can be deduced that $H_{t}(\alpha ,\beta ,\theta )$ is a Bernoulli variable with mean αβ/2, which implies that for any β∈[0,1], the sequence $\overset{\infty }{\underset{t=\text{1}}{\left\{H_{t}(\alpha ,\beta ,\theta )-\alpha \beta /\text{2}\right\}}}$ should also be an MDS. Using that $h_{t}(\alpha ,\beta ,\theta )=\text{1}\left(R_{j,t}\leq \overset{j}{\underset{\alpha ,t}{VaR}}\right)*\text{1}\left(R_{i,t}\leq \overset{i|j}{\underset{\beta |\alpha ,t}{CoVaR}}\right)$, $H_{t}$ can be computed using the following formula:


$$\begin{array}{c} \begin{array}{c} \begin{array}{c} H_{t}(\alpha ,\beta ,\theta )=\frac{\text{1}}{\beta }*\text{1}\left(R_{j,t}\leq \overset{j}{\underset{\alpha ,t}{VaR}}\right)*\int_{\text{0}}^{\beta }\text{1}\left(R_{i,t}\leq \overset{i|j}{\underset{\beta |\alpha ,t}{CoVaR}}\right)dz\\ =\frac{\text{1}}{\beta }\text{1}\left(u_{j,t}\leq \alpha \right)*\int_{\text{0}}^{\beta }\text{1}\left(u_{i|j,t}\leq z\right)dz\\ =\frac{\text{1}}{\beta }\left(\beta -u_{i|j,t}\right)\text{1}\left(u_{i|j,t}\leq \beta \right)\text{1}\left(u_{j,t}\leq \alpha \right) \end{array} \end{array} \end{array}$$ (16)

where $u_{j,t}=F_{j,t}\left(R_{j,t}\right)$ denotes the cumulative distribution function for $R_{j,t}$, and $u_{i|j,t}=F_{i|j,t}\left(R_{i,t}\right)$ represents the cumulative distribution function for $R_{i,t}$ under the condition $R_{j,t}\leq \overset{j}{\underset{\alpha ,t}{VaR}}$. Both sequences $\overset{\infty }{\underset{t=\text{1}}{\left\{u_{j,t}\right\}}}$ and $\overset{\infty }{\underset{t=\text{1}}{\left\{u_{i|j,t}\right\}}}$ are independent and identically distributed sequences following a U[0,1] uniform distribution. Notably, $H_{t}$ is independent of the distribution forms for $R_{i,t}$ and $R_{j,t}$.

Then, the joint violation process $h_{t}$ is employed to construct the conditional coverage tests for CoVaR. If the CoVaR model is correctly specified, then the m-th order autocorrelation coefficients for the joint violation process $h_{t}$ are zero, that is $\rho_{\text{1}}=\rho_{\text{2}}=\ldots =\rho_{m}=\text{0}$:


$$\begin{array}{c} \begin{array}{c} \rho_{j}=\frac{\gamma_{j}(\theta )}{\gamma_{\text{0}}(\theta )}\;,\;\gamma_{j}(\theta )=\frac{\text{1}}{T-j}\sum_{t=\text{1}+j}^{T}\left(h_{t}(\alpha ,\beta ,\theta )-\alpha \beta \right)\left(h_{t-j}(\alpha ,\beta ,\theta )-\alpha \beta \right) \end{array} \end{array}$$ (17)

Then, the following statistic for the conditional coverage test is derived:


$$\begin{array}{c} \begin{array}{c} {IND}_{CoVaR}(m)=T\sum_{j=\text{1}}^{m}{\hat{\rho_{j}}}^{\text{2}} \end{array} \end{array}$$ (18)

where $\hat{\rho_{j}}=\frac{\hat{\gamma_{j}}(\hat{\theta })}{\hat{\gamma_{\text{0}}}(\hat{\theta })}$, $\hat{\gamma_{j}}(\hat{\theta })\;=\frac{\text{1}}{T-j}\sum_{t=\text{1}+j}^{T}\left(h_{t}\left(\alpha ,\beta ,\hat{\theta }\right)-\alpha \beta \right)\left(h_{t-j}\left(\alpha ,\beta ,\hat{\theta }\right)-\alpha \beta \right)$. Under certain assumptions, ${IND}_{CoVaR}(m)$ converges to a chi-squared distribution with m degrees of freedom.

The cumulative joint violation process $H_{t}$ is employed to construct the conditional coverage tests for CoES. See Appendix for detailed proofs and Monte Carlo simulations of the conditional coverage tests for CoES. If the CoES model is correctly specified, the m-th order autocorrelation coefficients for the cumulative joint violation process $H_{t}$ are zero, that is $\rho_{\text{1}}=\rho_{\text{2}}=\ldots =\rho_{m}=\text{0}$.


$$\begin{array}{c} \begin{array}{c} \rho_{j}=\frac{\gamma_{j}(\theta )}{\gamma_{\text{0}}(\theta )}\;,\;\gamma_{j}(\theta )=\frac{\text{1}}{T-j}\sum_{t=\text{1}+j}^{T}\left(H_{t}(\alpha ,\beta ,\theta )-\alpha \beta /\text{2}\right)\left(H_{t-j}(\alpha ,\beta ,\theta )-\alpha \beta /\text{2}\right) \end{array} \end{array}$$ (19)

From this, the following conditional coverage test statistic is constructed:


$$\begin{array}{c} \begin{array}{c} {IND}_{CoES}(m)=T\sum_{j=\text{1}}^{m}{\hat{\rho_{j}}}^{\text{2}} \end{array} \end{array}$$ (20)

where $\hat{\rho_{j}}=\frac{\hat{\gamma_{j}}(\hat{\theta })}{\hat{\gamma_{\text{0}}}(\hat{\theta })}$, $\hat{\gamma_{j}}(\hat{\theta })\;=\frac{\text{1}}{T-j}\sum_{t=\text{1}+j}^{T}\left(H_{t}\left(\alpha ,\beta ,\hat{\theta }\right)-\alpha \beta /\text{2}\right)\left(H_{t-j}\left(\alpha ,\beta ,\hat{\theta }\right)-\alpha \beta /\text{2}\right)$. Under certain assumptions, ${IND}_{CoES}(m)$ converges to a chi-squared distribution with m degrees of freedom.

The logic of the cumulative joint violation process $H_{t}$ is best understood by starting from the standard univariate VaR backtest and extending it along two dimensions. The first dimension is cumulative: moving from VaR to ES backtesting. In a VaR backtest, one simply counts violations—days when the realized loss exceeds the predicted VaR. ES backtesting goes further by asking whether, on violation days, the magnitude of the loss is consistent with the predicted ES. This requires tracking the cumulative sum of scaled violations over time, rather than merely their frequency. The second dimension is joint: moving from VaR to CoVaR backtesting. Instead of asking whether one institution experiences a violation, we ask whether, conditional on institution i being in distress, the loss of institution j exceeds the predicted CoVaR. This defines a joint violation—a co-exceedance of two thresholds across two institutions. Our CoES framework naturally combines these two extensions: we construct a cumulative joint violation process that tracks, over time, whether the co-exceedances between institution pairs are consistent with the predicted CoES, both in frequency and in serial dependence.

### 2.4. Network estimation

When constructing the tail risk networks, the most common approach in previous studies is employed, ΔCoVaR and ΔCoES, to depict the directed risk spillover relationships. Specifically, as $\Delta \overset{i|j}{\underset{\beta |\alpha ,t}{CoVaR}}$ and $\overset{i|j}{\underset{\beta |\alpha ,t}{\Delta CoES}}$ are directional, $\Delta \overset{i|j}{\underset{\beta |\alpha ,t}{CoVaR}}$ (or $\Delta \overset{i|j}{\underset{\beta |\alpha ,t}{CoES}}$) does not necessarily equal $\Delta \overset{j|i}{\underset{\alpha |\beta ,t}{CoVaR}}$ (or $\Delta \overset{j|i}{\underset{\alpha |\beta ,t}{CoES}}$) when i ≠ j. $\Delta \overset{i|j}{\underset{\beta |\alpha ,t}{CoVaR}}$ is defined as:


$$\begin{array}{c} \begin{array}{c} \Delta \overset{i|j}{\underset{\beta |\alpha ,t}{CoVaR}}=\overset{i|j}{\underset{\beta |\alpha ,t}{CoVaR}}-\overset{i|j}{\underset{\beta |median,t}{CoVaR}} \end{array} \end{array}$$ (21)

where $\overset{i|j}{\underset{\beta |median,t}{CoVaR}}$ represents the VaR for institution i when institution j is in a normal state. The normal state is defined as $\overset{j}{\underset{\text{0}.\text{25},t}{VaR}}\leq R_{j,t}\leq \overset{j}{\underset{\text{0}.\text{75},t}{VaR}}$, where $\overset{j}{\underset{\text{0}.\text{25},t}{VaR}}$ and $\overset{j}{\underset{\text{0}.\text{75},t}{VaR}}$ respectively denote the 25% and 75% quantiles of the random variable $R_{j,t}$. $\Delta \overset{i|j}{\underset{\beta |\alpha ,t}{CoVaR}}$ captures the net risk spillover from institution j to institution i.

Similarly, $\Delta \overset{i|j}{\underset{\beta |\alpha ,t}{CoES}}$ is defined as:


$$\begin{array}{c} \begin{array}{c} \Delta \overset{i|j}{\underset{\beta |\alpha ,t}{CoES}}=\overset{i|j}{\underset{\beta |\alpha ,t}{CoES}}-\overset{i|j}{\underset{\beta |median,t}{CoES}} \end{array} \end{array}$$ (22)

$\Delta \overset{i|j}{\underset{\beta |\alpha ,t}{CoES}}$ similarly characterizes the risk spillover from institution j to institution i. Using the high-dimensional dynamic Factor Copula model, $\Delta \overset{i|j}{\underset{\beta |\alpha ,t}{CoVaR}}$ and $\overset{i|j}{\underset{\beta |\alpha ,t}{\Delta CoES}}$ are estimated for each period t, where i ≠ j. To clarify the primary risk spillover relationships in the tail risk network, a thresholding method is employed to filter out smaller ΔCoVaR and ΔCoES values. Comparing the ΔCoVaR (or ΔCoES) from the same institution at different times t, we need to determine a percentile threshold to filter out smaller values, thereby establishing the threshold for each i|j relationship. Specifically, we establish a functional relationship between the percentile threshold and the global efficiency of the network, then $\overset{i|j}{\underset{CoVaR}{Threshold}}$ and $\overset{i|j}{\underset{CoES}{Threshold}}$ are selected by monitoring the knee point of the curve. Global efficiency $E_{global}$ of a network G with N nodes is defined as:


$$\begin{array}{c} \begin{array}{c} E_{global}=\frac{\text{1}}{N(N-\text{1})}\sum_{i\neq j}\frac{\text{1}}{d_{ij}} \end{array} \end{array}$$ (23)

where $d_{ij}$ denotes the shortest path length between nodes i and j, defined as the minimum number of edges that must be traversed to go from i to j.

Knee-point detection is a heuristic method for selecting a threshold in complex network construction. It identifies a point that balances network sparsity and topological integrity by locating a pronounced change in the slope of a network metric (e.g., global efficiency) plotted against threshold values. The primary advantage of using the knee point of global efficiency, rather than those of network density or information entropy, for threshold selection lies in its direct optimization of a core functional property—the network’s capacity for parallel information integration. Unlike density, which only reflects connection sparsity without topological insight, or entropy, which emphasizes structural heterogeneity rather than functional performance, global efficiency captures the cost–efficiency trade-off intrinsic to many real-world networks. Following this rationale, we apply the knee-point detection method to the global efficiency curve of our tail-risk network to determine a percentile threshold. This empirically derived threshold is then used to filter the smaller ΔCoVaR and ΔCoES values. For each pair (i, j) at time t, an adjacency matrix element $\overset{i|j}{\underset{t}{a}}$ is determined by setting it to 1 if the spillover value is greater than or equal to the threshold, and to 0 otherwise:


$$\begin{array}{c} \text{CoVaR network}:\overset{i|j}{\underset{t}{a}}=\left\{ \begin{array}{c} @c\text{1},\overset{i|j}{\underset{CoVaR}{\text{Threshold}}}\leq \Delta \overset{i|j}{\underset{\beta |\alpha ,t}{CoVaR}}\\ \text{0},otherwise \end{array}\right.\; \end{array}$$

$$\begin{array}{c} \begin{array}{c} @c\text{CoES network}:\overset{i|j}{\underset{t}{a}}=\left\{ \begin{array}{c} @c\text{1},\overset{i|j}{\underset{CoES}{\text{Threshold}}}\leq \Delta \overset{i|j}{\underset{\beta |\alpha ,t}{CoES}}\\ \text{0},otherwise \end{array}\right.\; \end{array} \end{array}$$ (24)

Filtering risk measures into 0 or 1 using the thresholding method allows for a comparison of the overall risk spillover (out-degree) and overall risk contagion (in-degree) of different institutions in the same period t. A higher network density indicates the retention of a greater number of significant risk interaction relationships during that period. Finally, through the adjacency matrix $A_{t}$ shown in formula 25, a directed and unweighted tail risk network is constructed based on the $\Delta \overset{i|j}{\underset{\beta |\alpha ,t}{CoVaR}}$ and $\Delta \overset{i|j}{\underset{\beta |\alpha ,t}{CoES}}$ risk spillover relationships.


$$\begin{array}{c} \begin{array}{c} A_{t}=\;\begin{array}{cccccc} \; & I_{\text{1}} & I_{\text{2}} & I_{\text{3}} & \ldots & I_{N}\\ I_{\text{1}} & \text{0} & \overset{\text{2}|\text{1}}{\underset{t}{a}} & \overset{\text{3}|\text{1}}{\underset{t}{a}} & \ldots & \overset{N|\text{1}}{\underset{t}{a}}\\ I_{\text{2}} & \overset{\text{1}|\text{2}}{\underset{t}{a}} & \text{0} & \overset{\text{3}|\text{2}}{\underset{t}{a}} & \ldots & \overset{N|\text{2}}{\underset{t}{a}}\\ I_{\text{3}} & \overset{\text{1}|\text{3}}{\underset{t}{a}} & \overset{\text{2}|\text{3}}{\underset{t}{a}} & \text{0} & \ldots & \overset{N|\text{3}}{\underset{t}{a}}\\ \vdots & \vdots & \vdots & \vdots & \ddots & \vdots \\ I_{N} & \overset{\text{1}|N}{\underset{t}{a}} & \overset{\text{2}|N}{\underset{t}{a}} & \overset{\text{3}|N}{\underset{t}{a}} & \ldots & \text{0} \end{array} \end{array} \end{array}$$ (25)

## 3. Results

### 3.1. Data

This study uses weekly return data for 55 Chinese A-share listed financial institutions from January 1, 2011, to December 31, 2022, covering 580 trading weeks. The data are sourced from the China Stock Market & Accounting Research (CSMAR) database. Given the quasi-financial characteristics of the real estate sector in China, this study incorporates real estate companies into the analysis as financial institutions [28]. The real estate sector, a cornerstone of China’s economy for two decades, accounts for approximately 39% of total bank lending. Additionally, substantial capital from bond markets, equity investments, and trust products flows into this sector. Our sample represents approximately 80% of the total market capitalization across financial and real estate sectors, based on China Securities Regulatory Commission (CSRC) industry classifications. This comprehensive coverage includes five subsectors: (1) banking, (2) securities, (3) insurance, (4) other financial services, and (5) real estate (see Table 1).

**Table 1:** Abbreviations and full names for the 55 A-listed Chinese financial institutions.

	Real estate		Bank
WK	China Vanke	ICBC	Industrial and Commercial Bank of China
BL	Poly Developments and Holdings	CBC	China Construction Bank Corporation
HXXF	China Fortune Land Development	ABC	Agricultural Bank of China
HQC	Shenzhen Overseas Chinese Town	BOC	Bank of China
JDJT	Gemdale Corporation	CMB	China Merchants Bank
LJZ	Shanghai Lujiazui Finance & Trade Zone Development	BOCM	Bank of Communications
RSFZ	Risesun Real Estate Development	IBC	Industrial Bank
FHKG	China Oceanwide Holdings	SPDB	Shanghai Pudong Development Bank
XHZB	Xinhu Zhongbao	CITB	China CITIC Bank
TH	Thaihot Group	CMCB	China Minsheng Banking
ZTJR	Zhongtian Financial Group Company	CEB	China Everbright Bank
YGC	Yango Group	PAB	Ping An Bank
YGE	Youngor Group	BOB	Bank of Beijing
JRJ	Financial Street Holdings	HXB	Hua Xia Bank
JKGF	Jinke Property Group	BNB	Bank of Ningbo
ZNJS	Jiangsu Zhongnan Construction Group	BNJ	Bank of Nanjing
	Security		Insurance
CITS	CITIC Securities	PAIC	Ping An Insurance Company of China
HASC	Haitong Securities	CLIC	China Life Insurance Company
CNPC	CNPC Capital	CPIC	China Pacific Insurance
GFSC	GF Securities	TMIC	Tianmao Industry
HTSC	Huatai Securities		Other
CMSC	China Merchants Securities	MMCC	Minmetals Capital
EBSC	Everbright Securities	AXTC	Anxin Trust
SDIC	SDIC Capital	SITC	Shaanxi International Trust
IDSC	Industrial Securities	JDTZ	Kunwu Jiuding Investment Holdings
CJSC	Changjiang Securities	XMJK	Panda Financial Holdings
GYSC	Guoyuan Securities	MSKG	Minsheng Holdings
GZYX	Guangzhou Yuexiu Capital Holdings	AJJT	Shanghai AJ Group

### 3.2. Network backtesting results

We backtest the tail risk networks to validate the proposed Factor-Copula-CoES model. Using the backtesting frameworks for CoVaR and CoES described in Section 2.3, we test each edge of the tail-risk network and aggregate the results. In assessing the network backtesting results, we employ the ‘rejection rate’ indicator, defined as the proportion of network edges that fail the backtest. A lower rejection rate suggests better model adequacy, implying that the underlying CoVaR/CoES estimation model is more appropriate for constructing the tail risk network.

**Fig 1:**
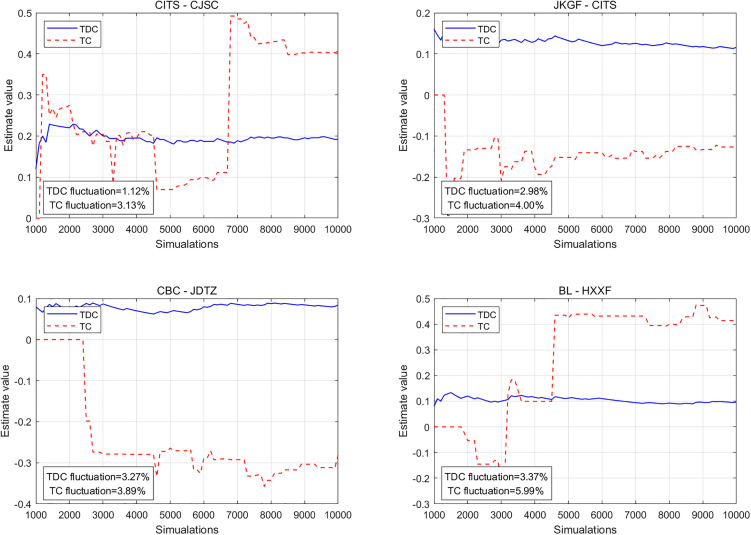
TDC and TC convergence diagnostics.

For comparison, the backtesting results of tail risk networks constructed using the bivariate static Copula model and the bivariate dynamic Copula model are also examined. Specifically, nine bivariate static Copula models: Normal, Student t, Gumbel, Rotated Gumbel, Clayton, Rotated Clayton, Frank, Plackett, and SJC; and four bivariate dynamic Copula models: time-varying Normal, time-varying Student t, time-varying Rotated Gumbel, and time-varying SJC; are considered. AIC and BIC criteria are used to determine the optimal bivariate static and dynamic Copula models. For the bivariate (static and dynamic) copula models, CoVaR and CoES are estimated using standard parametric methods. For the high-dimensional factor copula model, these measures are estimated via simulation, with 10,000 repetitions per estimation. To ensure the reliability of our simulation-based estimates for the factor copula model, we verify the convergence of key dependence measures. Since accurate estimation of CoVaR and CoES depends on accurately capturing tail dependence, we conduct convergence diagnostics on the tail dependence coefficient (TDC) and tail correlation (TC) derived from the simulated data. TDC quantifies the asymptotic probability of simultaneous extreme events, whereas TC measures the strength of dependence in the distribution tails. For illustration, we randomly selected four institution pairs—(CITS, CJSC), (JKGF, CITS), (CBC, JDTZ), and (BL, HXXF)—and examine the convergence of TDC and TC for these pairs in the 200th trading week (see Fig 1). The fluctuations of TDC and TC were computed over the final 2,000 simulation runs. Following common practice in simulation studies, we consider TDC and TC to have converged when their variations over the final 2,000 runs fall below thresholds of 0.05 and 0.08, respectively. As illustrated in the figure, all four institutional pairs show clear convergence at 10,000 simulations, and the convergence patterns observed for other randomly sampled pairs are similar.

Tables 2 and 3 present the backtesting results for tail-risk networks constructed using the CoVaR and CoES measures, respectively. In each table, Panel A reports results for the network built with the Factor Copula model, Panel B for the bivariate dynamic copula model, and Panel C for the bivariate static copula model.

**Table 2:** Average backtest p-values and rejection rates for the CoVaR tail risk network.

		Panel A: Factor copula		Panel B: Time-varying copula		Panel C: Static copula	
${IND}_{CoVaR}(m)$		IND(5)	IND(10)	IND(5)	IND(10)	IND(5)	IND(10)
Real Estate	P value	0.9493	0.8537	0.4097	0.3981	0.3530	0.3403
	Rejection rate	4.98%	12.62%	59.03%	60.19%	64.70%	65.97%
Bank	P value	0.8994	0.8638	0.4468	0.4448	0.3391	0.3357
	Rejection rate	10.07%	13.43%	55.32%	55.44%	66.09%	66.44%
Securities	P value	0.9606	0.9135	0.4676	0.4676	0.3858	0.3844
	Rejection rate	3.86%	6.33%	53.24%	53.24%	61.42%	61.42%
Insurance	P value	0.9176	0.8145	0.5000	0.5000	0.4583	0.4583
	Rejection rate	8.33%	14.81%	50.00%	50.00%	54.17%	54.17%
Others	P value	0.9116	0.8553	0.3471	0.3435	0.2169	0.2011
	Rejection rate	8.47%	8.99%	64.81%	65.34%	78.31%	79.89%
Overall	P value	0.9301	0.8670	0.4317	0.4273	0.3465	0.3394
	Rejection rate	6.90%	11.18%	56.77%	57.21%	65.35%	66.03%

IND(m) is the m-th order statistic for conditional coverage test. CoVaR’s confidence level α = β = 0.05. For reporting convenience, the report industry-level averages for p-value and rejection rate are reported.

**Table 3:** Average backtest p-values and rejection rates for the CoES tail risk network.

		Panel A: Factor copula		Panel B: Time-varying copula		Panel C: Static copula	
${IND}_{CoES}(m)$		IND(5)	IND(10)	IND(5)	IND(10)	IND(5)	IND(10)
Real Estate	P value	0.9442	0.8869	0.4097	0.4019	0.3542	0.3430
	Rejection rate	4.98%	10.07%	59.03%	59.61%	64.58%	65.51%
Bank	P value	0.8910	0.8715	0.4479	0.4456	0.3391	0.3378
	Rejection rate	9.87%	12.12%	55.21%	55.44%	66.09%	66.20%
Securities	P value	0.9600	0.9355	0.4685	0.4691	0.3870	0.3859
	Rejection rate	3.24%	4.78%	53.09%	53.09%	61.27%	61.27%
Insurance	P value	0.9315	0.8971	0.5000	0.5000	0.4583	0.4583
	Rejection rate	4.17%	7.87%	50.00%	50.00%	54.17%	54.17%
Others	P value	0.9195	0.8826	0.3456	0.3453	0.2169	0.2142
	Rejection rate	6.88%	8.99%	64.81%	64.81%	78.31%	78.31%
Overall	P value	0.9281	0.8932	0.4320	0.4292	0.3471	0.3428
	Rejection rate	6.21%	9.22%	56.70%	56.94%	65.29%	65.59%

IND(m) is the m-th order statistic for conditional coverage test. CoES’s confidence level α = β = 0.05. For reporting convenience, the report industry-level averages for p-value and rejection rate are reported.

Panel A of Table 2 shows that the Factor-Copula-CoVaR network has conditional coverage rejection rates of 6.9% and 11.18% at autocorrelation orders 5 and 10, respectively. Notably, the rejection rates are higher for edges involving banking institutions. The results in Panels B and C suggest that both the bivariate dynamic and static Copula models achieve conditional coverage rejection rates exceeding 50%, which is inferior to the Factor-Copula-CoVaR model. The CoES conditional coverage test results in Table 3 corroborate these findings: the high-dimensional Factor Copula model consistently yields lower rejection rates than both bivariate dynamic and static copula models in constructing CoES-based tail-risk networks.

As shown in Panel A of Tables 2 and 3, the Factor-Copula-CoES model exhibits somewhat lower rejection rates in IND(5) and IND(10) tests than its CoVaR counterpart. To systematically evaluate the performance of the CoES-based models and verify whether the observed differences are statistically significant, we conduct McNemar tests against a set of benchmark models (including the Static-copula-CoES and Time-varying-copula-CoES). A significant result indicates that the two models differ significantly in their backtesting outcomes, and the model with a lower rejection rate can be considered superior. Table 4 reports the results. The findings show that the Factor-Copula-CoES model yields significantly lower rejection rates than both the Static-copula-CoES and Time-varying-copula-CoES models under the IND(5) and IND(10) metrics. Compared with the Factor‑Copula‑CoVaR model, however, the Factor‑Copula‑CoES model does not show a significant advantage in rejection rate under the IND(5) metric, whereas it remains significantly superior under the IND(10) metric. The proposed factor‑copula‑CoES model reduces the IND(10) rejection rates by 56.37%, 47.72%, and 1.96% relative to the static‑copula‑CoES, time‑varying‑copula‑CoES, and factor‑copula‑CoVaR models, respectively, with all reductions being statistically significant. Given CoES’s superior ability to characterize tail loss correlations and its robust performance in backtests, we select the Factor-Copula-CoES model for constructing China’s financial system’s high-dimensional dynamic tail risk network. In addition, to assess whether our tail-risk network construction methodology is robust to the choice of data frequency, we replicate the entire analysis at a daily frequency. The results, detailed in Appendix D, confirm that the core network architecture is consistently captured across frequencies, while also revealing the effects of microstructure noise inherent in daily returns.

**Table 4:** McNemar test: model comparison of Factor-Copula-CoES and alternative models.

	Factor-Copula-CoES	
	IND(5)	IND(10)
Static-copula-CoES	0.000***	0.000***
	(1692.95)	(1548.47)
Time-varying-copula-CoES	0.000***	0.000***
	(1443.81)	(1308.29)
Factor-Copula-CoVaR	0.919	0.002***
	(0.01)	(9.14)

This table reports the p-values of the McNemar test, with the McNemar’s chi-squared statistic shown in parentheses. *** indicates statistical significance at the 1% level, implying that the two models yield significantly different rejection rates in the tail risk network backtesting.

### 3.3. Dynamic analysis of the tail risk network

This section constructs a high-dimensional dynamic tail-risk network for China’s financial system (comprising 55 A-share listed institutions) using the selected Factor-Copula-CoES model. To characterize the levels of risk spillover and contagion, we employ topological network analysis to extract key systemic risk features. These include overall network characteristics, node-specific metrics (with particular attention to out-degree and in-degree), and their time-varying trends.

**Fig 2:**
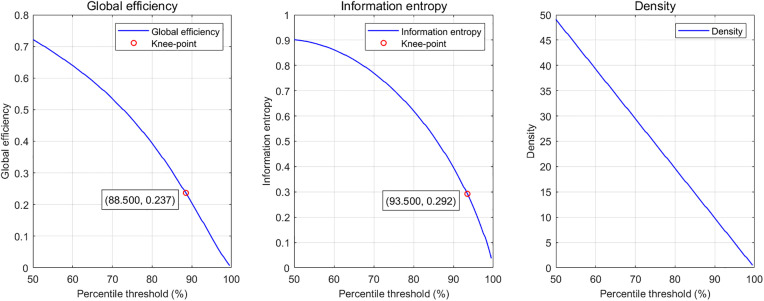
Evolution of network global efficiency, information entropy, and density under different percentile thresholds.

First, the knee-point detection method is employed to determine the network threshold. Fig 2 plots network density, information entropy, and global efficiency of the tail-risk network as functions of the percentile threshold. Because the network examined in this paper is dynamic, all metrics are reported as weekly averages. In general, as the percentile threshold rises, network density, information entropy, and global efficiency all show a monotonic decreasing trend. Both the information‑entropy curve and the global‑efficiency curve exhibit mathematical knee points. The knee point of the information‑entropy curve corresponds to a percentile threshold of 93.5%, while that of the global‑efficiency curve corresponds to a percentile threshold of 88.5%. Given that the primary objective of our tail-risk network analysis is to capture systemic risk propagation—a process reliant on efficient information transmission—we prioritize the threshold identified by the global-efficiency curve (88.5%). This choice is functionally grounded. Global efficiency directly quantifies a network’s integration capacity, which is crucial for understanding risk propagation speed. In contrast, while information entropy provides insights into structural heterogeneity, it does not have a direct bearing on dynamic spillover processes.

**Fig 3:**
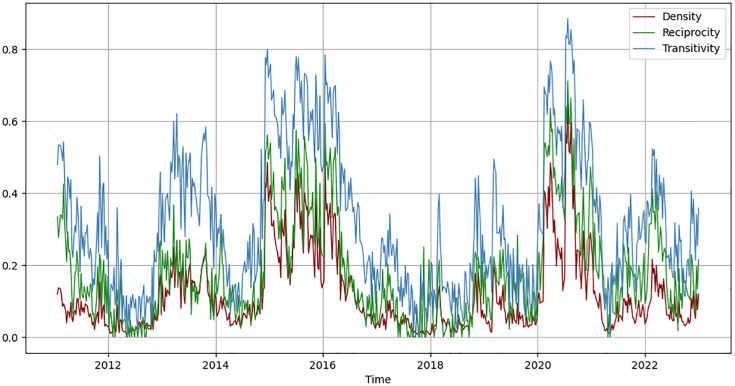
Network density, reciprocity, and transitivity trends from 2011 to 2022.

As outlined in Section 2.4, once the threshold is determined, we construct a directed, unweighted tail‑risk network based on the CoES measure. We examine the dynamic evolution of the tail-risk network’s overall characteristics to assess systemic risk across the financial system. Fig 3 displays the weekly trends of network density, transitivity, and reciprocity from 2011 to 2022. These three metrics demonstrate synchronized dynamics and comparable clustering patterns. Notably, all metrics show significant elevations during three crisis periods: (1) the 2013 interbank liquidity crisis, (2) the 2015–2016 stock market collapse, and (3) the 2020 COVID-19 pandemic – each corresponding to major financial turbulence in China. The June 2013 interbank market liquidity crunch triggered severe banking system stress. During 2015–2016, China’s stock market experienced unprecedented volatility, with frequent “limit-down” events (when stocks hit the maximum allowed daily decline) eroding nearly 50% of total market capitalization by June 2016. The 2020 pandemic then transmitted substantial macroeconomic shocks to the financial sector. The responsive fluctuations in our network metrics confirm that the high-dimensional dynamic tail-risk network effectively captures regime shifts in China’s systemic risk profile.

**Fig 4:**
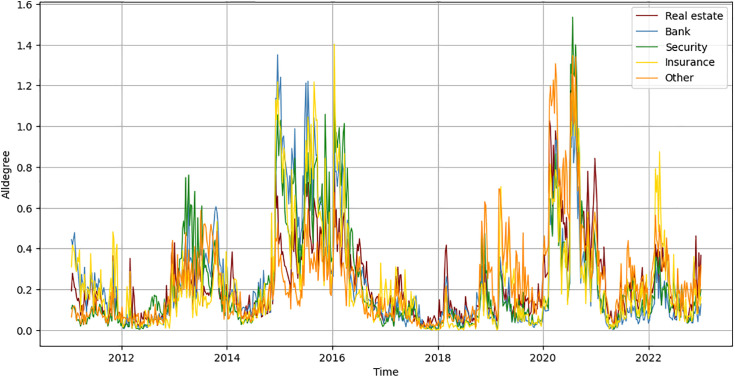
Node all-degree trends for each sector from 2011 to 2022.

**Fig 5:**
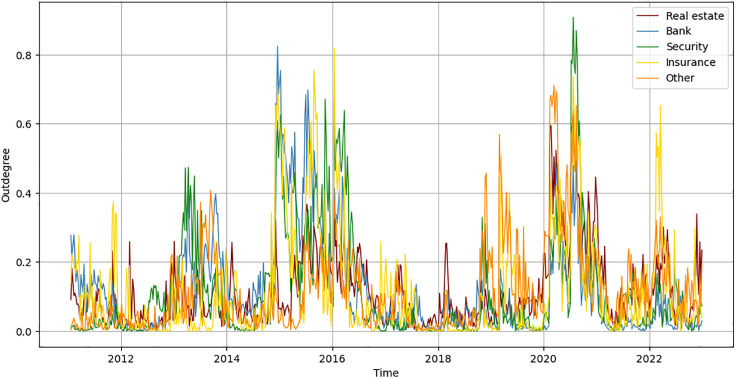
Node out-degree trends in each sector from 2011 to 2022.

**Fig 6:**
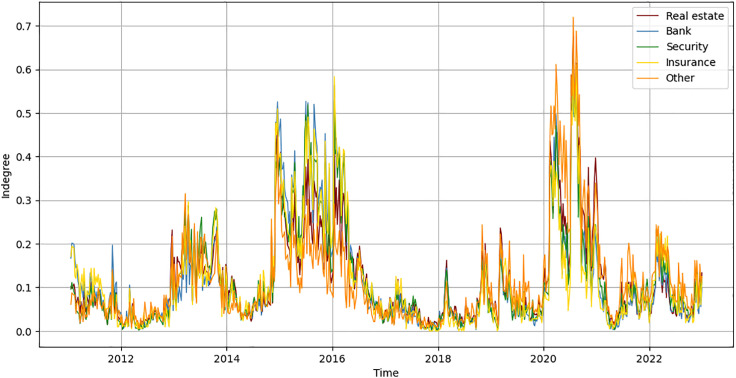
Node in-degree trends in each sector from 2011 to 2022.

We next analyze the dynamic evolution of nodal characteristics in the tail-risk network: all-degree (total connections), out-degree (risk spillover), and in-degree (risk contagion). Figs 4-6 present the weekly trends of these metrics, with industry averages shown for clarity. The main observations from Figs 4–6 are as follows. (1) Industry-average all-degree, out-degree, and in-degree all show pronounced peaks during the three crisis periods: 2013, 2015–2016, and 2020. (2) On average, out-degree exceeds in-degree across industries. While the evolution of in-degree is relatively consistent (homogeneous) across industries, out-degree trends display substantial heterogeneity. Consequently, the temporal pattern of all-degree closely mirrors that of out-degree, as the latter dominates the variation in total connections. (3) The temporal peaks of out-degree and in-degree (Figs 5 and 6) tend to concentrate in different industries at different times. This indicates that the epicenters of risk spillover and contagion shift across the financial sectors over the sample period.

**Fig 7:**
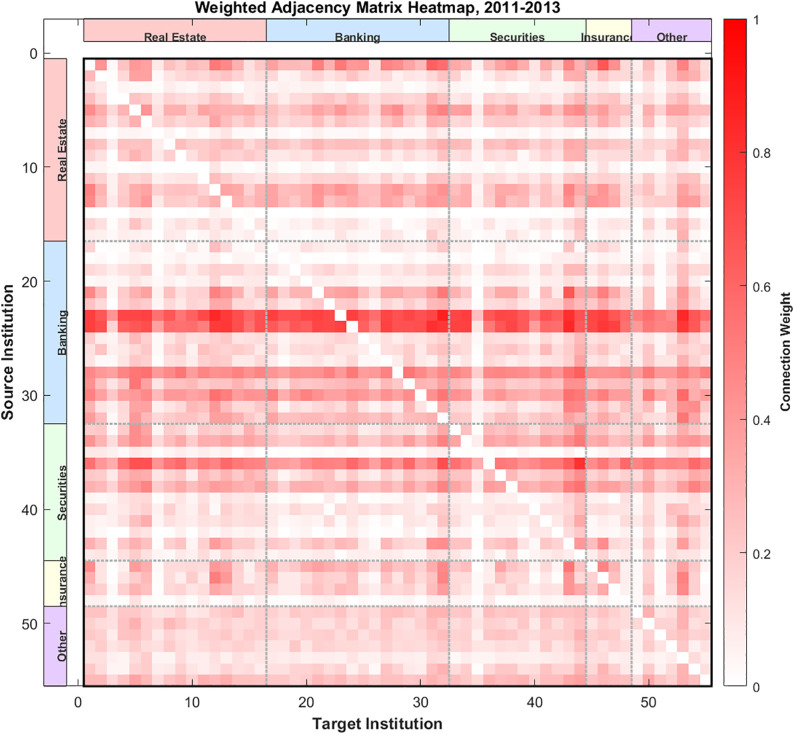
Weighted adjacency matrix of tail risk network (2011-2013).

**Fig 8:**
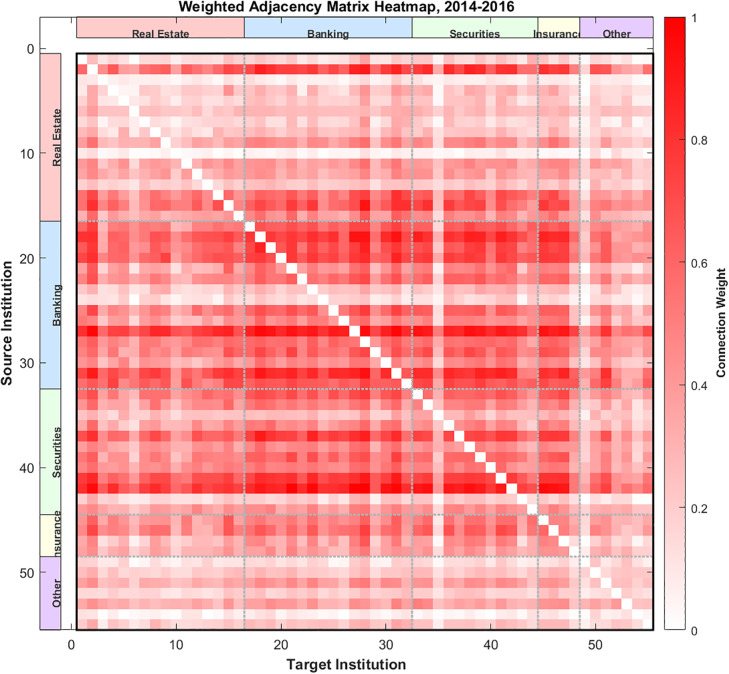
Weighted adjacency matrix of tail risk network (2014-2016).

**Fig 9:**
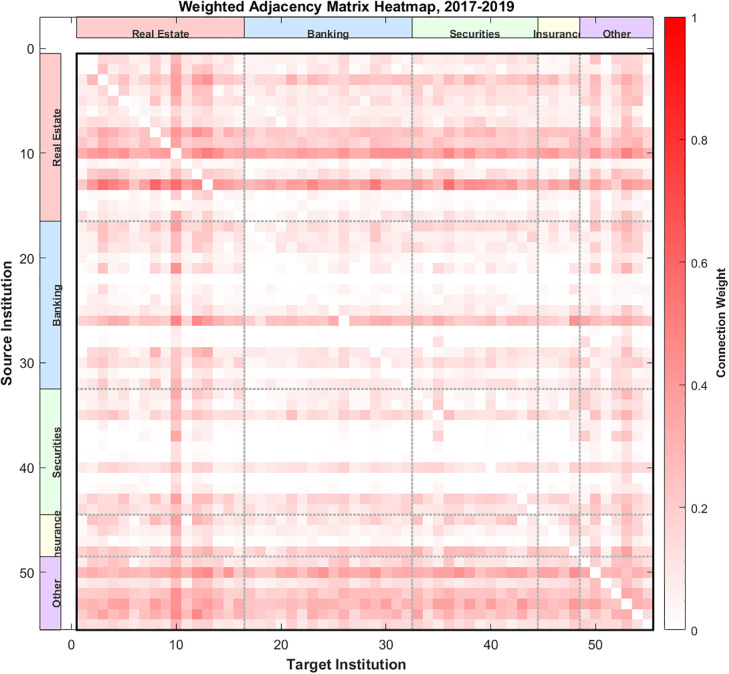
Weighted adjacency matrix of tail risk network (2017-2019).

**Fig 10:**
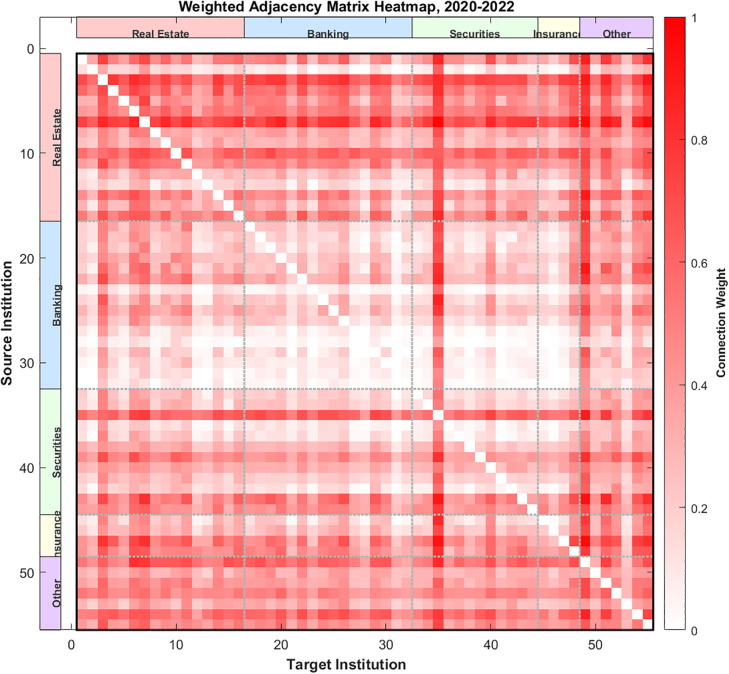
Weighted adjacency matrix of tail risk network (2020-2022).

To visualize the evolving dynamics of the tail-risk network, we divide the 12-year sample into four distinct periods (see Figs 7–10): 2011–2013, 2014–2016, 2017–2019, and 2020–2022. Our analysis reveals two salient temporal patterns. First, the tail-risk networks are markedly denser during the 2014–2016 and 2020–2022 sub-periods. This heightened connectivity coincides with two major systemic-risk events in China: the stock market crash (2015–2016) and the COVID-19 pandemic (2020–2022). Second, the core of the risk network shifts notably across periods. During 2014–2016, risk spillovers were centered predominantly in the banking and securities sectors. In contrast, the 2020–2022 period saw real estate and other financial sectors become the primary foci of risk.

To clearly illustrate the risk spillover and contagion across different financial institutions during China’s various risk events, the financial institutions are classified and ranked based on their cumulative out-degrees and in-degrees to identify the systemically important financial institutions (SIFIs). Three distinct risk periods are identified: June 2013 is characterized as a ‘cash crunch’, 2015−2016 as a ‘stock market crash’, and 2020−2022 as the ‘COVID-19 pandemic’. Tables 5–7 show the financial institution rankings in these three risk periods. Table 5 shows that during the ‘cash crunch’, the primary contributors to the risk spillovers are securities firms. Table 6 shows that during the ‘stock market crash’ period, the tail risk spillovers and contagion are mainly concentrated in the banking sectors. Notably, bank BNB ranks among the top three in both out-degree and in-degree, positioning it as a potentially critical nexus for risk transmission within the network during this period. Table 7 indicates that during the ‘COVID-19 pandemic’, the risk spillovers are mainly associated with some real estate institutions, and the risk contagion is associated with real estate and other financial institutions. Similarly, real estate firm HXXF appears as a key player, ranking highly in both measures, suggesting its central role in risk propagation during the pandemic.

**Table 5:** Top 10 financial institutions ranked based on in-degree and out-degree sums during the ‘cash crunch’ period.

Rank	Abbreviation	Industry	In_sum	Abbreviation	Industry	Out_sum
1	GZYX	Security	1.48	HTSC	Security	3.78
2	JDJT	Real estate	1.30	CMSC	Security	3.19
3	MSKG	Other	1.30	CITS	Security	2.98
4	GYSC	Security	1.24	HUSC	Security	2.69
5	LJZ	Real estate	1.13	GFSC	Security	2.37
6	AJJT	Other	1.13	MMCC	Other	2.35
7	AXTC	Other	1.07	CMCB	Bank	1.76
8	SPDB	Bank	1.04	CEB	Bank	1.74
9	BNJ	Bank	1.04	YGC	Real estate	1.61
10	GFSC	Security	1.02	SPDB	Bank	1.56

**Table 6:** Top 10 financial institutions ranked based on the in-degree and out-degree sums during the ‘stock market crash’ period.

Rank	Abbreviation	Industry	In_sum	Abbreviation	Industry	Out_sum
1	PAB	Bank	31.87	CEB	Bank	46.80
2	BNB	Bank	28.70	BNB	Bank	41.00
3	BL	Real estate	27.98	CBC	Bank	39.48
4	IDSC	Security	27.72	HUSC	Security	37.94
5	IBC	Bank	27.30	BOC	Bank	34.72
6	CBC	Bank	27.26	JKGF	Real estate	33.89
7	CEB	Bank	26.78	JRJ	Real estate	33.70
8	CMSC	Security	26.59	ABC	Bank	33.69
9	EBSC	Security	26.48	CITS	Security	32.48
10	CMB	Bank	25.63	IDSC	Security	31.39

**Table 7:** Top 10 financial institutions ranked based on the in-degree and out-degree sums during the ‘COVID-19’ period.

Rank	Abbreviation	Industry	In_sum	Abbreviation	Industry	Out_sum
1	CNPC	Security	32.37	HXXF	Real estate	39.85
2	HXXF	Real estate	29.93	RSFZ	Real estate	39.31
3	MMCC	Other	29.02	HQC	Real estate	32.57
4	RSFZ	Real estate	26.93	MSKG	Other	32.24
5	MSKG	Other	26.70	ZTJR	Real estate	27.98
6	AJJT	Other	26.07	JRJ	Real estate	27.94
7	SITC	Other	23.89	CPIC	Insurance	27.65
8	LJZ	Real estate	22.98	JDTZ	Other	26.93
9	JDTZ	Other	22.94	ZNJS	Real estate	26.46
10	ZTJR	Real estate	22.89	WK	Real estate	25.41

## 4. Network systemic risk determinants

The topological analysis of the tail‑risk network offers a valuable perspective for risk decomposition, enabling us to distinguish between two distinct channels of systemic risk: risk spillover and risk contagion. Unlike conventional systemic‑risk studies that rely on CoVaR (or CoES) metrics, this framework allows for a clear separation of spillover and contagion effects. In this section, we develop an econometric model to examine the determinants of tail systemic risk in Chinese financial institutions and to differentiate how various factors influence risk contagion and risk spillover. Specifically, we estimate an individual fixed‑effects panel regression in which institutional and market determinants of systemic risk serve as explanatory variables, and the total degree, out‑degree, and in‑degree centrality of financial institutions in the tail‑risk network serve as dependent variables:


$$\begin{array}{cc} y_{i,t}=\; & \beta_{\text{0}}+\beta_{\text{1}}{size}_{i,t-k}+\beta_{\text{2}}{leverage}_{i,t-k}+\;\beta_{\text{3}}{mismatch}_{i,t-k}+\beta_{\text{4}}{beta}_{i,t-k}+\beta_{\text{5}}{vol}_{i,t-k}\\ & \;\;\;+\beta_{\text{6}}{ES}_{i,t-k}+\beta_{\text{7}}{dib}_{t-k}+\beta_{\text{8}}{liq}_{t-k}+\beta_{\text{9}}{dtr}_{t-k}+\;\beta_{\text{10}}{dco}_{t-k}+\beta_{\text{11}}{dgo}_{t-k}+\beta_{\text{12}}{hs\text{300}}_{t-k}\\ & \;\;\;+\beta_{\text{13}}{vix}_{t-k}\;+\mu_{i}+\varepsilon_{i,t} \end{array}$$

where $y_{i,t}$ is the all-degree, out-degree, and in-degree of financial institution i in the tail risk network in week t; μ is the individual fixed effects; and k is the explanatory variable lag, which is set at 1, 2, 3, and 4 in subsequent discussions to examine the term structure of the influencing factors. Drawing from Adrian and Brunnermeier [4], as shown in Table 8, six institutional-level variables and seven market-level variables are selected as the explanatory variables. The data for the institutional and market-level explanatory variables are sourced from the Wind database, the institutional-level variables; size, leverage, and mismatch; are quarterly data and the remainder are weekly data. Table 8 shows the definitions and descriptive statistics for the explanatory variables.

**Table 8:** Definitions and summary statistics for the independent variables.

Variables	Definition	Mean	Sd	Min	Max
size	log of the total assets	26.3200	2.6532	19.7036	31.3086
leverage	ratio of total debt to total assets	0.7596	0.2080	0.0085	1.0182
mismatch	ratio of non-cash short-term debt to total debt	0.3245	0.4287	−0.2269	1.0000
beta	beta for the rolling CAPM model	1.0619	0.4050	0.0848	3.0423
vol	equity return volatility	0.3575	0.2037	0.0000	2.4737
ES	expected shortfall	0.1202	0.0614	0.0064	1.2590
dib	change in the three-month SHIBOR rate	0.0009	1.1760	−4.6000	4.2510
liq	difference between the three-month SHIBOR rate and the three-month repo rate	1.7461	0.9417	−0.1712	5.5805
dtr	spread between the 10-year government bond yield and the three-month Treasury bill rate	0.8181	0.3683	−1.5118	1.8795
dco	credit spread between the 10-year AA-rated corporate bond and the 10-year government bond	2.3582	0.5505	1.2876	3.6416
dgo	credit spread between the 10-year local government financing vehicle bond and the government bond	2.4865	0.8280	1.0285	4.4326
hs300	H&S300 Composite Index returns	0.0004	0.0305	−0.1402	0.1066
vix	annualized daily volatility in the H&S300 Index	0.2038	0.0955	0.0442	0.7164

Table 9 shows the regression results for the impact of the institutional and market variables on the financial institution’s node all-degree; columns 1–4 respectively lag the explanatory variables by 1, 2, 3, and 4 quarters. Table 9 indicates that the ‘size’ variable consistently exerted a significant positive influence on the institutional network all-degree in all model specifications and implies that the larger institutions tended to have higher systemic risk, which was consistent with existing research [4]. However, the ‘leverage’ variable negatively impacted the network all-degree, which indicated that institutions with higher leverage possibly had reduced systemic risk, which is a different finding from previous international studies [13]. The ‘mismatch’ variable, which indicates an asset-liability duration mismatch, was not found to significantly influence systemic risk, which was possibly because of the relatively low overall duration mismatch in Chinese financial institutions. The stock market price-based variables ‘beta’, ‘vol’, and ‘ES’ all significantly and positively affected institutional systemic risk, which was as expected. The influence of the ‘beta’ regression coefficient remained stable in the different lag quarters, the impact of ‘vol’ grew, and the impact of ‘ES’ decreased.

**Table 9:** Panel regression results for the dependent all-degree variable.

	(1)	(2)	(3)	(4)
Variables	All-degree 1-quarter	All-degree 2-quarter	All-degree 3-quarter	All-degree 4-quarter
size	0.037***	0.037***	0.039***	0.039***
	(4.42)	(4.47)	(4.38)	(4.28)
leverage	−0.109***	−0.111***	−0.118***	−0.122***
	(−2.81)	(−2.74)	(−2.77)	(−2.76)
mismatch	0.001	0.007	0.013	0.016
	(0.03)	(0.34)	(0.56)	(0.70)
beta	0.134***	0.139***	0.143***	0.147***
	(6.73)	(6.71)	(6.61)	(6.58)
vol	0.042	0.150***	0.240***	0.309***
	(1.01)	(3.54)	(5.04)	(5.86)
ES	1.908***	1.455***	1.047***	0.723***
	(13.62)	(11.07)	(8.07)	(5.48)
dib	0.009	0.011**	0.014**	0.014*
	(1.63)	(2.21)	(2.30)	(1.68)
liq	−0.011	−0.010	−0.017*	−0.024*
	(−1.42)	(−1.47)	(−1.84)	(−1.81)
dtr	−0.048	−0.041	−0.030	−0.020
	(−1.60)	(−1.35)	(−0.95)	(−0.60)
dco	0.196***	0.227***	0.248***	0.260***
	(3.27)	(3.54)	(3.77)	(3.85)
dgo	−0.173***	−0.191***	−0.201***	−0.206***
	(−4.63)	(−4.68)	(−4.72)	(−4.68)
hs300	0.527	0.316	0.572*	0.610**
	(1.53)	(0.79)	(1.70)	(1.98)
vix	1.030***	0.896***	0.806***	0.723***
	(7.46)	(6.45)	(5.92)	(5.43)
Constant	−1.246***	−1.240***	−1.284***	−1.301***
	(−5.23)	(−5.28)	(−5.13)	(−5.00)
Observations	31,185	31,130	31,075	31,020
R-squared	0.343	0.302	0.281	0.266
Number of groups	55	55	55	55

t-statistics reported in parentheses are based on the Driscoll-Kraay standard errors allowing for autocorrelation, heteroscedasticity, and cross-sectional dependence. ***, ** and * indicate that the statistics are significant at the 1%,5% and 10% levels, respectively.

At the market level, the variables representing market interest rate changes (‘dib’), corporate credit risk (‘dco’), and overall market volatility (‘vix’) all had a significantly positive influence on the network all-degree. The ‘dco’ variable impact intensified over the lag quarters, the ‘vix’ variable impact decreased, and the ‘dgo’ variable, which represented local government credit risk, had a significant negative impact on the network all-degree, which was not in line with theoretical expectations. As the ‘dgo’ variable indicates the spread between local government financing vehicle bonds and national bonds, a higher ‘dgo’ indicates higher local government bond interest rates, increased financing pressure, or higher local government debt risk. Theoretically, local government debt risk should amplify institutional systemic risk. However, the regression results suggest that the higher ‘dgo’ values correspond to reduced systemic risk. These counterintuitive patterns regarding the liability variable and the ‘dgo’ variable likely stem from the unique institutional structure of China’s financial system, which differs substantially from the mature markets that underpin standard theoretical predictions. We discuss these issues further in the “Future Research” section at the end of the article.

Table 10 shows the varying impacts of the institutional and market-level factors on the institutions’ risk spillovers and contagion. The first three columns show the outcomes when the network in-degree is taken as the dependent variable and the explanatory variables are lagged by 1–3 quarters, and the subsequent three columns show the results when the network out-degree is taken as the dependent variable and the explanatory variables are similarly lagged.

**Table 10:** Panel regression results for the dependent in-degree and out-degree variables.

	(1)	(2)	(3)	(4)	(5)	(6)
Variables	In-degree 1-quarter	In-degree 2-quarter	In-degree 3-quarter	Out-degree 1-quarter	Out-degree 2-quarter	Out-degree 3-quarter
size	0.014***	0.014***	0.015***	0.023***	0.023***	0.023***
	(3.04)	(3.11)	(3.15)	(5.37)	(5.41)	(5.22)
leverage	−0.064***	−0.065***	−0.067***	−0.045**	−0.047**	−0.051**
	(−3.31)	(−3.21)	(−3.16)	(−1.99)	(−1.96)	(−2.06)
mismatch	0.017	0.019	0.020	−0.016	−0.011	−0.008
	(1.38)	(1.52)	(1.63)	(−1.32)	(−0.85)	(−0.52)
beta	0.047***	0.048***	0.049***	0.088***	0.091***	0.094***
	(5.13)	(5.21)	(5.15)	(7.41)	(7.29)	(7.19)
vol	−0.007	0.007	0.019	0.050*	0.143***	0.221***
	(−0.40)	(0.38)	(0.97)	(1.78)	(4.87)	(6.69)
ES	−0.048	−0.068	−0.092*	1.956***	1.523***	1.140***
	(−1.05)	(−1.50)	(−1.90)	(17.61)	(14.91)	(11.49)
dib	0.005*	0.006**	0.007**	0.004	0.005**	0.007**
	(1.69)	(2.20)	(2.28)	(1.54)	(2.18)	(2.29)
liq	−0.005	−0.005	−0.008*	−0.006	−0.005	−0.009*
	(−1.29)	(−1.30)	(−1.71)	(−1.55)	(−1.63)	(−1.96)
dtr	−0.032*	−0.028*	−0.022	−0.016	−0.013	−0.008
	(−1.96)	(−1.66)	(−1.24)	(−1.10)	(−0.92)	(−0.57)
dco	0.131***	0.147***	0.157***	0.064**	0.081***	0.091***
	(3.91)	(4.13)	(4.30)	(2.36)	(2.72)	(3.01)
dgo	−0.109***	−0.117***	−0.121***	−0.064***	−0.075***	−0.080***
	(−5.25)	(−5.25)	(−5.20)	(−3.71)	(−3.88)	(−4.02)
hs300	0.333*	0.223	0.347*	0.194	0.093	0.225
	(1.80)	(1.05)	(1.92)	(1.19)	(0.48)	(1.42)
vix	0.746***	0.669***	0.613***	0.284***	0.228***	0.193***
	(10.19)	(9.26)	(8.85)	(4.19)	(3.26)	(2.75)
Constant	−0.413***	−0.428***	−0.465***	−0.833***	−0.813***	−0.818***
	(−3.19)	(−3.31)	(−3.39)	(−6.69)	(−6.63)	(−6.29)
Observations	31,185	31,130	31,075	31,185	31,130	31,075
R-squared	0.358	0.321	0.304	0.312	0.266	0.239
Number of groups	55	55	55	55	55	55

t-statistics reported in parentheses are based on the Driscoll-Kraay standard errors allowing for autocorrelation, heteroscedasticity, and cross-sectional dependence. ***, ** and * indicate that the statistics are significant at the 1%,5% and 10% levels, respectively.

The comparison of the network out-degree and in-degree regression results in Table 10 reveals that the ‘size’ variable significantly influences both the out-degree and in-degree, with the impact on the out-degree being more pronounced. This indicates that an institution’s scale simultaneously amplifies its risk contagion and risk spillovers, especially the latter. As institution size increases, the resilience to risks also rises; therefore, the impact of size on risk contagion is lower than the impact on risk spillover.

The ‘leverage’ variable has a significant negative impact on the in-degree but does not significantly influence the out-degree at a 1% confidence level. This suggests that an increase in institutional leverage can reduce its risk contagion. The ‘beta’ variable significantly affects both the out-degree and in-degree, with a greater impact on the out-degree. Because ‘beta’ represents the undiversified risks in an institution’s portfolio, it has been traditionally seen as a systemic risk indicator. These results confirm that ‘beta’ significantly influences the tail risk levels of the institutions. While the institutional variables ‘vol’ and ‘ES’ both have positive, significant impacts on the individual institution’s out-degrees, their influence on the in-degree is inconsequential. This suggests that even though an institution’s return volatility (‘vol’) and individual tail risk can increase their tail risk spillover, they do not significantly affect the tail risk contagion.

When the market-level variables’ effects on the in-degree and out-degree are compared, the direction and significance of the regression coefficients remain consistent, but their magnitudes differ. The increase in corporate credit risk (‘dco’), the escalation in market volatility (‘vix’), and the reduction in the central government’s implicit guarantee expectations (‘dgo’) all result in significantly positive impacts on risk contagion and spillover, with the influence on risk contagion being more potent than on risk spillover.

In summary, the institutional and market-level factors are found to have different influences on the institutions’ in-degree and out-degree, which highlights the varying risk factor impacts on risk contagion and spillover in financial institutions, that is, institutional-level factors have a stronger influence on risk spillover, but market-level factors have a greater impact on risk contagion. This distinction aligns with the systemic risk literature, where spillover (out-degree) refers to shock propagation through fundamental linkages, and contagion (in-degree) denotes vulnerability to tail events transmitted from distressed peers—a phenomenon that intensifies during crises [4,29]. Our finding that institutional factors primarily drive spillover while market factors shape contagion complements existing evidence on the distinct determinants of systemic risk transmission and vulnerability.

## 5. Conclusion and future research

This study seeks to estimate the tail risk measures, CoVaR and CoES, in high-dimensional scenarios using the Factor Copula model and validate the efficacy of the tail risk measure estimations through backtesting. Based on the tail risk measure relationships, a dynamic high-dimensional tail risk network is constructed. Previous research on tail risk network construction, which has been limited to indirectly estimating tail risk measures using quantile regression models, has failed to effectively capture the intricate non-linear dependencies between institutions and was not conducive to backtesting. The approach taken in this study improves upon these deficits by incorporating the Factor Copula model and constructing a comprehensive framework for unconditional and conditional backtests for CoVaR and CoES. Based on the backtesting results, an optimal Factor-Copula-CoES model is employed to construct a tail risk network that comprises Chinese banking, real estate, securities, insurance and other financial industries. Using network topology analyses, rich dynamic network characteristics, such as network density and node degree, are extracted. The dynamic network analysis results align with significant Chinese financial market risk events. The quantitative analyses reveal that institutional and market-level factors have varying impacts on institutional in-degrees and out-degrees; they suggest that institutional-level factors have a stronger effect on risk spillover, whereas market-level factors are more influential in driving risk contagion. This study aids regulators and investors in better understanding the systemic finance risk evolution in China and the factors that can influence risk spillover and contagion in financial institutions.

Future research may follow three directions: specificity studies of China’s financial market, construction of multi‑layer tail‑risk networks, and the introduction of graph neural network (GNN)‑based artificial intelligence algorithms.

Two interesting anomalies emerge in the present study of tail‑risk determinants: higher leverage and higher dgo (local government financing vehicle bond spread) are associated with lower systemic risk levels of institutions. According to conventional financial theory, leverage typically represents an institution’s default risk, and the dgo signals local‑government default risk; both should be positively correlated with systemic risk. The anomalies, however, may be explained by the specific institutional context of China’s financial market. In China’s bank‑dominated indirect‑financing system, higher leverage may reflect stronger financing capacity, thereby reducing systemic risk. As for the dgo variable, because local government bonds carry an implicit central‑government guarantee, a rising spread may shift systemic risk toward the central fiscal authority, thus lowering the systemic risk borne by financial institutions. By identifying suitable instrumental variables for institutional financing capacity and the central government’s implicit guarantee, future studies could design mediation‑effect tests to formally examine the anomalies described above.

The tail‑risk network constructed in this paper is a lower‑tail network based on CoES(i|j) relationships. Future studies could build multi‑layer tail‑risk networks by altering the edge‑formation mechanism or by constructing upper‑tail risk networks. For instance, edge relationships could be defined based on the similarity of CoES measures across nodes, which may better capture risk resonance and diversification. Moreover, combining upper‑ and lower‑tail networks would provide a more comprehensive characterization of systemic tail risk. These ideas open new possibilities for constructing multi‑layer tail‑risk networks.

The dynamic tail‑risk network constructed here also creates opportunities for introducing graph‑based artificial intelligence algorithms such as graph neural networks (GNNs). In recent years, GNNs have advanced rapidly, owing to their strong ability to mine graph‑structured information and their high accuracy in classification and prediction tasks, and have been widely adopted in transportation, computer science, and social‑network analysis. However, their application in financial risk research faces two major challenges: correlation‑based models often suffer from the “curse of dimensionality,” and GNN‑type algorithms typically require large volumes of data. The high‑dimensional dynamic tail‑risk network proposed in this paper provides rich feature data for GNNs and allows them to fully utilize their network‑information mining capabilities. The integration of graph neural networks with tail‑risk networks shows considerable promise in financial domains such as systemic risk identification and prediction, as well as portfolio optimization.

## Supporting information

S1 Appendix Proofs and simulations of CoES backtesting. (DOCX)

S2 File Data and code used in this article. (ZIP)

## References

[1] Du Z, Escanciano JC. Backtesting Expected Shortfall: Accounting for Tail Risk. Management Science. 2017;63(4):940–58. doi: 10.1287/mnsc.2015.2342

[2] Acerbi C, Tasche D. On the coherence of expected shortfall. Journal of Banking & Finance. 2002;26(7):1487–503. doi: 10.1016/s0378-4266(02)00283-2

[3] Kratz M, Lok YH, McNeil AJ. Multinomial VaR backtests: A simple implicit approach to backtesting expected shortfall. Journal of Banking & Finance. 2018;88:393–407. doi: 10.1016/j.jbankfin.2018.01.002

[4] Adrian T, Brunnermeier MK. CoVaR. American Economic Review. 2016;106(7):1705–41.

[5] Barnett WA, Wang X, Xu H-C, Zhou W-X. Hierarchical contagions in the interdependent financial network. Journal of Financial Stability. 2022;61:101037. doi: 10.1016/j.jfs.2022.101037

[6] Brunetti C, Harris JH, Mankad S. Networks, interconnectedness, and interbank information asymmetry. Journal of Financial Stability. 2023;67:101163. doi: 10.1016/j.jfs.2023.101163

[7] Hautsch N, Schaumburg J, Schienle M. Financial Network Systemic Risk Contributions. Review of Finance. 2014;19(2):685–738. doi: 10.1093/rof/rfu010

[8] Härdle WK, Wang W, Yu L. TENET: Tail-Event driven NETwork risk. Journal of Econometrics. 2016;192(2):499–513. doi: 10.1016/j.jeconom.2016.02.013

[9] Chen CY-H, Härdle WK, Okhrin Y. Tail event driven networks of SIFIs. Journal of Econometrics. 2019;208(1):282–98. doi: 10.1016/j.jeconom.2018.09.016

[10] Bonaccolto G, Caporin M, Maillet BB. Dynamic large financial networks via conditional expected shortfalls. European Journal of Operational Research. 2022;298(1):322–36. doi: 10.1016/j.ejor.2021.06.037

[11] Girardi G, Tolga Ergün A. Systemic risk measurement: Multivariate GARCH estimation of CoVaR. Journal of Banking & Finance. 2013;37(8):3169–80. doi: 10.1016/j.jbankfin.2013.02.027

[12] Reboredo JC, Ugolini A. Systemic risk in European sovereign debt markets: A CoVaR-copula approach. Journal of International Money and Finance. 2015;51:214–44. doi: 10.1016/j.jimonfin.2014.12.002

[13] Karimalis EN, Nomikos NK. Measuring systemic risk in the European banking sector: a copula CoVaR approach. The European Journal of Finance. 2017;24(11):944–75. doi: 10.1080/1351847x.2017.1366350

[14] Oh DH, Patton AJ. Time-Varying Systemic Risk: Evidence From a Dynamic Copula Model of CDS Spreads. Journal of Business & Economic Statistics. 2017;36(2):181–95. doi: 10.1080/07350015.2016.1177535

[15] Opschoor A, Lucas A, Barra I, van Dijk D. Closed-Form Multi-Factor Copula Models With Observation-Driven Dynamic Factor Loadings. Journal of Business & Economic Statistics. 2020;39(4):1066–79. doi: 10.1080/07350015.2020.1763806

[16] Chang J, Hao X, Chen Z. Research on structural breaks in dependence: dynamic factor Copula models. Applied Economics Letters. 2025;:1–8. doi: 10.1080/13504851.2025.2566399

[17] Chen Z, Zhou J, Hao X. Dynamic factor copula-based modeling for market risk optimization with an application to the real industry in China. Journal of Innovation & Knowledge. 2023;8(4):100453. doi: 10.1016/j.jik.2023.100453

[18] Escanciano JC, Pei P. Pitfalls in backtesting Historical Simulation VaR models. Journal of Banking & Finance. 2012;36(8):2233–44. doi: 10.1016/j.jbankfin.2012.04.004

[19] Du Z, Pei P. Backtesting portfolio value‐at‐risk with estimated portfolio weights. Journal Time Series Analysis. 2020;41(5):605–19. doi: 10.1111/jtsa.12524

[20] Banulescu-Radu D, Hurlin C, Leymarie J, Scaillet O. Backtesting Marginal Expected Shortfall and Related Systemic Risk Measures. Management Science. 2021;67(9):5730–54. doi: 10.1287/mnsc.2020.3751

[21] van Oordt M, Zhou C. Systemic risk and bank business models. J of Applied Econometrics. 2018;34(3):365–84. doi: 10.1002/jae.2666

[22] De Jonghe O, Diepstraten M, Schepens G. Banks’ size, scope and systemic risk: What role for conflicts of interest?. Journal of Banking & Finance. 2015;61:S3–13. doi: 10.1016/j.jbankfin.2014.12.024

[23] Tabak BM, Fazio DM, Cajueiro DO. Systemically important banks and financial stability: The case of Latin America. Journal of Banking & Finance. 2013;37(10):3855–66. doi: 10.1016/j.jbankfin.2013.06.003

[24] Brunnermeier MK, Oehmke M. The Maturity Rat Race. The Journal of Finance. 2013;68(2):483–521. doi: 10.1111/jofi.12005

[25] Silva AF. Strategic Liquidity Mismatch and Financial Sector Stability. The Review of Financial Studies. 2019;32(12):4696–733. doi: 10.1093/rfs/hhz044

[26] Beltratti A, Stulz RM. The credit crisis around the globe: Why did some banks perform better?. Journal of Financial Economics. 2012;105(1):1–17. doi: 10.1016/j.jfineco.2011.12.005

[27] Sklar A. Fonctions de répartition à n dimensions et leurs marges. Publications del’Institut de Statistique de L’Université de Paris. 1959;8:229–31.

[28] Bellavite Pellegrini C, Cincinelli P, Meoli M, Urga G. The contribution of (shadow) banks and real estate to systemic risk in China. Journal of Financial Stability. 2022;60:101018. doi: 10.1016/j.jfs.2022.101018

[29] Forbes KJ, Rigobo R. No Contagion, Only Interdependence: Measuring Stock Market Comovements. The Journal of Finance. 2002;57:2223–61.

