# Supplementary material for: Dynamic financial tail risk networks: A backtesting-based conditional expected shortfall approach
Source: PLoS One. 2026 Jun 24;21(6):e0351966. doi: 10.1371/journal.pone.0351966 (PMC13293451; doi:10.1371/journal.pone.0351966)
Supplement: S1 Appendix — (DOCX) [file pone.0351966.s001.docx]

**Appendix A. Assumptions**

To prove the asymptotic properties of the proposed backtest statistic for CoES, we introduce the following assumptions A0-A4. Let $\parallel\cdot\parallel$ denote the Euclidean norm, and let $C$ be a generic constant that may change from expression to expression, and let $\Theta_{0}$ be an arbitrary neighborhood of $\theta_{0}\in\Theta$.

Assumption A0: The conditional distribution of $Y_{i,t}$ given $\Omega_{t-1}$ is $F_{i}\left( \cdot\mid\theta_{0},\Omega_{t-1} \right)$, and the cumulative distribution function of $Y_{j,t}$ given $Y_{i,t}\leq-VaR_{i,t}\left( \alpha,\theta_{0} \right)$ is $F_{Y_{j,t}\mid Y_{i,t}\leq-VaR_{i,t}\left( \alpha,\theta_{0} \right)}\left( \cdot\mid\theta_{0},\Omega_{t-1} \right)$.

Assumption A1: $\left\{ Y_{t},X_{t} \right\}_{t=-T+1}^{n}$ Is strictly stationary and ergodic.

Assumption A2: The estimator $\theta_{T}$ is $\sqrt{T}$-consistent estimator for $\theta_{0}$, where $\theta_{0}$ is in the interior of $\Theta$, and $\theta_{T}$ satisfies the following asymptotic Bahadur expansion, $\sqrt{T}\left( \theta_{T}-\theta_{0} \right)=\frac{1}{\sqrt{T}}\sum_{t=-T+1}^{0} l_{t}+o_{p}\left( 1 \right),$ where $l_{t}$ satisfies $E\left( l_{t}\mid\Omega_{t-1} \right)=0,$ and $\sum_{0}$ exists and is positive definite.

Assumption A3: $E\left[ H_{t}(\alpha,\beta,\theta)\mid\Omega_{t-1} \right]$ is continuously differentiable in $\theta$, and

$$E\left[ \sup_{\theta\in\Theta_{0}} \left\| \frac{\partial E\left[ H_{t}(\alpha,\beta,\theta)\mid\Omega_{t-1} \right]}{\partial\theta} \right\| \right]<C$$

Assumption A4: $n\to\infty,T\to\infty,\lambda:=limn/T=0$ .

Assumption A0 is commonly used in the literature, which assumes that the model is correctly specified. The assumption A1 is for ease of exposition, our results can also be extended to some non-stationary and non-ergodic sequences. Assumption A2 is satisfied by most commonly used estimators, such as the (quasi-) maximum likelihood estimator and the generalized method of moments estimator. Assumption A3 is required for the asymptotic equicontinuity of certain empirical processes and the uniform law of large numbers.

**Appendix B. Proofs**

Proof of the Conditional Coverage Test ${IND}_{COES}$ :

Under Assumptions A0-A3 we obtain the following argument,

$$\begin{matrix} & \frac{1}{\sqrt{n-j}}\sum_{t=1+j}^{n} \left[ H_{t}-E\left( H_{t}\mid\Omega_{t-1} \right) \right]\left( H_{t-j}-\frac{\alpha\beta}{2} \right) \\ & -\frac{1}{\sqrt{n-j}}\sum_{t=1+j}^{n} \left[ H_{t}-E\left( H_{t}\mid\Omega_{t-1} \right) \right]\left( H_{t-j}-\frac{\alpha\beta}{2} \right)=o_{p}(1) \end{matrix}$$

Therefore,

$$\begin{matrix} & \sqrt{n-j}\left( \gamma_{j}\left( \theta_{T} \right)-\gamma_{j}\left( \theta_{0} \right) \right) \\ & =\frac{1}{\sqrt{n-j}}\sum_{t=1+j}^{n} \left[ E\left( H_{t}\mid\Omega_{t-1} \right)-\alpha\beta/2 \right]\left( H_{t-j}-\frac{\alpha\beta}{2} \right)- \\ & \frac{1}{\sqrt{n-j}}\sum_{t=1+j}^{n} \left[ E\left( H_{t}\mid\Omega_{t-1} \right)-\alpha\beta/2 \right]\left( H_{t-j}-\frac{\alpha\beta}{2} \right)+o_{p}(1) \\ & =\frac{1}{\sqrt{n-j}}\sum_{t=1+j}^{n} \left[ E\left( H_{t}\mid\Omega_{t-1} \right)-E\left( H_{t}\mid\Omega_{t-1} \right) \right]\left( H_{t-j}-\frac{\alpha\beta}{2} \right)+o_{p}(1) \\ & =\sqrt{\lambda}\sqrt{T}\left( \theta_{T}-\theta_{0} \right)^{'}\frac{1}{n-j}\sum_{t=1+j}^{n} \frac{\partial E\left[ H_{t}\left( \alpha,\beta,\theta_{T} \right)\mid\Omega_{t-1} \right]}{\partial\theta}\left( H_{t-j}-\frac{\alpha\beta}{2} \right)+o_{p}(1) \\ & =\sqrt{\lambda}\sqrt{T}\left( \theta_{T}-\theta_{0} \right)^{'}\frac{\partial\gamma_{j}\left( \theta_{0} \right)}{\partial\theta}+o_{p}(1) \end{matrix}$$

It can be further obtained that

$$\begin{matrix} \sqrt{n-j}\left( \rho_{j}\left( \theta_{T} \right)-\rho_{j}\left( \theta_{0} \right) \right) & =\frac{\sqrt{n-j}\left( \gamma_{nj}-\gamma_{nj} \right)}{\alpha\beta(1/3-\alpha\beta/4)}+o_{p}(1) \\ & =R_{j}^{'}\sqrt{\lambda}\sqrt{T}\left( \theta_{T}-\theta_{0} \right)+o_{p}(1) \end{matrix}$$

Therefore,

$$\begin{matrix} \sqrt{n-j}\rho_{j}\left( \theta_{T} \right)=\sqrt{n-j}\rho_{j}\left( \theta_{0} \right)+R_{j}^{'}\sqrt{\lambda}\sqrt{T}\left( \theta_{T}-\theta_{0} \right)+o_{p}(1) \\ =\frac{1}{\sqrt{n-j}\alpha\beta(1/3-\alpha\beta/4)}\sum_{t=1+j}^{n} \left( H_{t}-\frac{\alpha\beta}{2} \right)\left( H_{t-j}-\frac{\alpha\beta}{2} \right)+ \\ R_{j}^{'}\sqrt{\lambda}\frac{1}{\sqrt{T}}\sum_{t=-T+1}^{0} l_{t}+o_{p}(1) \end{matrix}$$

Consider $\sqrt{n}\left( \rho_{1}\left( \theta_{0} \right),\rho_{2}\left( \theta_{0} \right)\cdots\rho_{m}\left( \theta_{0} \right) \right)^{'}\to^{d}N\left( 0,I_{m} \right)$, and since the first term on the right-hand side of the above equation $\sqrt{n-j}\rho_{j}\left( \theta_{0} \right)$ and the second term of the right-hand side of the above equation $R_{j}^{'}\sqrt{\lambda}\sqrt{T}\left( \theta_{T}-\theta_{0} \right)$ are summed over out-of-sample and in-sample observations, respectively, so the covariance between these two terms is zero.

Thus, we have $\sqrt{n}\rho^{(m)}\to^{d}N\left( 0,\sum\right)$. Where, the $\mathrm{ij}$-th element of $\sum$ is $\sum_{ij} =\delta_{ij}+\lambda R_{i}^{'}\sum_{0} R_{j}$, $R_{j}=\frac{1}{\alpha\beta(1/3-\alpha\beta/4)}\frac{\partial\gamma_{j}\left( \theta_{0} \right)}{\partial\theta}，\delta_{ij}$ is the Kronecker delta function, which takes the value 1 if i＝j and 0 otherwise.

This leads to the following asymptotic distribution of the conditional coverage test statistic ${IND}_{CoES}(m)$,

$${IND}_{CoES}(m)=n\sum_{j=1}^{m} \rho_{j}^{2}\left( \theta_{T} \right)\to^{d}\sum_{j=1}^{m} \pi_{j}Z_{j}^{2}$$

where, the $\left\{ \pi_{j} \right\}_{j=1}^{m}$ are the m eigenvalue of the matrix $\sum$, the $\left\{ Z_{j} \right\}$ are independent standard normal distribution variables.

In particular, when $\lambda=0$, there is $\sum_{ij} =\delta_{ij}$ , i.e. $\pi_{j}=1,j=1,\cdots,m$, as in this case

$${IND}_{CoES}(m)\to^{d}\chi_{m}^{2}$$

where $\chi_{m}^{2}$ is a chi-square distribution with m degrees of freedom.

**Appendix C. Monte Carlo simulations**

To assess the finite sample performance of the proposed backtest statistic for CoES, we carry out some Monte Carlo studies. Our null and alternative data generating processes for $\left\{ Y_{jt},Y_{it} \right\}$ are as follows, where the the subscript $j$ denotes the $j$-th financial institution, the subscript $i$ denotes the $i$-th financial institution.

$H_{0}$ : DCC-GJRGARCH $(1,1,1)$ Model .

$$\begin{matrix} & Y_{j,t}=\sigma_{j,t}\varepsilon_{j,t}, \\ & \sigma_{j,t}^{2}=0.01+\left( 0.01+0.1I_{j,t-1} \right)Y_{j,t-1}^{2}+0.93\sigma_{j,t-1}^{2}, \\ & Y_{i,t}=\sigma_{i,t}\varepsilon_{i,t}, \\ & \sigma_{i,t}^{2}=0.02+\left( 0.02+0.1I_{i,t-1} \right)Y_{i,t-1}^{2}+0.91\sigma_{i,t-1}^{2}, \\ & \varepsilon_{jit}=\left( \varepsilon_{j,t},\varepsilon_{i,t} \right)^{'}=R_{jit}^{1/2}z_{jit},z_{jit}\sim t_{5}, \\ & R_{jit}=diag\left( Q_{t} \right)^{-1/2}Q_{t}diag\left( Q_{t} \right)^{-1/2}, \\ & Q_{t}=0.03Q+0.03\left( \varepsilon_{jit-1}\varepsilon_{jit-1}^{'} \right)+0.94Q_{t-1}, \end{matrix}$$

where $t_{5}$ is the binary student-$t$ distribution with 5 degrees of freedom.

$A_{1}$ : DCC-TAR model.

$$\begin{matrix} Y_{j,t} & =a_{j,t}Y_{j,t-1}+\sigma_{j,t}\varepsilon_{j,t}, \\ a_{j,t} & =0.7\cdot1\left( \sigma_{j,t-1}\varepsilon_{j,t-1}\leq-2 \right), \\ \sigma_{j,t}^{2} & =0.04+0.1\sigma_{j,t-1}^{2}\varepsilon_{j,t-1}^{2}+0.89\sigma_{j,t-1}^{2}, \\ Y_{i,t} & =a_{i,t}Y_{i,t-1}+\sigma_{i,t}\varepsilon_{i,t}, \\ a_{i,t} & =0.7\cdot1\left( \sigma_{i,t-1}\varepsilon_{i,t-1}\leq-2 \right), \\ \sigma_{i,t}^{2} & =0.04+0.12\sigma_{i,t-1}^{2}\varepsilon_{i,t-1}^{2}+0.87\sigma_{i,t-1}^{2}, \end{matrix}$$

where, the $\varepsilon_{jit}=\left( \varepsilon_{j,t},\varepsilon_{i,t} \right)^{'}$ and $H_{0}$ are identical.

$A_{2}$ : DCC-EGARCH $(1,1)$ Model .

$Y_{j,t}=\sigma_{j,t}\varepsilon_{j,t}$,

$ln\sigma_{j,t}^{2}=0.01+0.9ln\sigma_{j,t-1}^{2}+0.3\left( \left| \varepsilon_{j,t-1} \right|-\sqrt{2/\pi} \right)-0.8\varepsilon_{j,t-1}$,

$Y_{i,t}=\sigma_{i,t}\varepsilon_{i,t}$,

$ln\sigma_{i,t}^{2}=0.01+0.91ln\sigma_{i,t-1}^{2}+0.28\left( \left| \varepsilon_{i,t-1} \right|-\sqrt{2/\pi} \right)-0.81\varepsilon_{i,t-1}$,

where, the $\varepsilon_{jit}=\left( \varepsilon_{j,t},\varepsilon_{i,t} \right)^{'}$ and $H_{0}$ are identical.

$A_{3}:DCC-ARCH(2)$ Model.

$$\begin{matrix} & Y_{j,t}=\sigma_{j,t}\varepsilon_{j,t}, \\ & \sigma_{j,t}^{2}=0.1+0.1\sigma_{j,t-1}^{2}\varepsilon_{j,t-1}^{2}+0.8\sigma_{j,t-2}^{2}\varepsilon_{j,t-2}^{2}, \\ & Y_{i,t}=\sigma_{i,t}\varepsilon_{i,t}, \\ & \sigma_{i,t}^{2}=0.1+0.12\sigma_{i,t-1}^{2}\varepsilon_{i,t-1}^{2}+0.78\sigma_{i,t-2}^{2}\varepsilon_{i,t-2}^{2}, \end{matrix}$$

where, the $\varepsilon_{jit}=\left( \varepsilon_{j,t},\varepsilon_{i,t} \right)^{'}$ and $H_{0}$ are identical.

$A_{4}:BEKK$ Model.

$Y_{t}=\left( Y_{j,t},Y_{i,t} \right)^{'}=H_{t}^{1/2}z_{t}$,

$H_{t}=C+A^{'}Y_{t-1}Y_{t-1}^{'}A+G^{'}Y_{t-1}^{*}Y_{t-1}^{*'}G+B^{'}H_{t-1}B$,

$Y_{t-1}^{*}=\left[ Y_{j,t}1\left( Y_{j,t}<0 \right),Y_{i,t}1\left( Y_{i,t}<0 \right) \right]^{'},z_{t}\sim t_{5}$,

$C=\left[ \begin{matrix} 0.01 & 0.0127 \\ 0.0127 & 0.01 \end{matrix} \right],A=\left[ \begin{matrix} 0.1 & 0 \\ 0 & \sqrt{0.02} \end{matrix} \right]$,

$$G=\left[ \begin{matrix} \sqrt{0.1} & 0 \\ 0 & \sqrt{0.1} \end{matrix} \right],B=\left[ \begin{matrix} \sqrt{0.93} & 0 \\ 0 & \sqrt{0.91} \end{matrix} \right]。$$

$A_{5}$ : GJRGARCH(1,1,1)-t Copula model.

$Y_{j,t}=\sigma_{j,t}\varepsilon_{j,t},\varepsilon_{j,t}\sim t_{5}$,

$\sigma_{j,t}^{2}=0.01+\left( 0.01+0.1I_{j,t-1} \right)Y_{j,t-1}^{2}+0.93\sigma_{j,t-1}^{2}$,

$Y_{i,t}=\sigma_{i,t}\varepsilon_{i,t},\varepsilon_{i,t}\sim t_{5}$,

$\sigma_{i,t}^{2}=0.02+\left( 0.02+0.1I_{i,t-1} \right)Y_{i,t-1}^{2}+0.91\sigma_{i,t-1}^{2}$,

$\left( F_{t_{5}}\left( \varepsilon_{j,t} \right),F_{t_{5}}\left( \varepsilon_{i,t} \right) \right)^{'}\sim t$ Copula $(0.8,1/0.15)$ ,

where $F_{t_{5}}$ is the student-$t$ cumulative distribution function with 5 degrees of freedom.

$A_{6}$ : DCC-GJRGARCH $(1,1,1)$ model, and the standardized error term $z_{jit}$ is subject to a mixed normal distribution. $Y_{j,t}、Y_{i,t}$ and $\varepsilon_{jit}$ are identical with $H_{0}$,

$$\begin{matrix} & z_{jit}\sim\left[ 0.6\cdot N\left( \mu_{1},\Sigma_{1} \right)+0.4\cdot N\left( \mu_{2},\Sigma_{2} \right) \right]/\sqrt{3} \\ & \mu_{1}=[1,1]^{'},\Sigma_{1}=\left[ \begin{matrix} 2 & 0 \\ 0 & 2 \end{matrix} \right], \\ & \mu_{2}=[-1.5,-1.5]^{'},\Sigma_{2}=\left[ \begin{matrix} 0.75 & 0 \\ 0 & 0.75 \end{matrix} \right] \end{matrix}$$

$A_{7}$ : DCC-GJRGARCH $(1,1,1)$ model, and the standardized error term $z_{jit}$ is subject to an asymmetric binary $t$ distribution. $Y_{j,t}、Y_{i,t}$ and $\varepsilon_{jit}$ are identical with $H_{0}$. The probability density function of $z_{jit}$ is as follows,

$$\begin{matrix} & f(z;v,\zeta)=\frac{C(v,\gamma)\cdot K_{1+v/2}\left( \sqrt{v+(z-\mu)^{'}\gamma^{-1}(z-\mu)\zeta^{'}\gamma^{-1}\zeta} \right)}{exp\left( -(z-\mu)^{'}\gamma^{-1}\zeta\right)} \\ & \times\frac{\left( 1+\frac{1}{v}(z-\mu)^{'}\gamma^{-1}(z-\mu) \right)^{-1-v/2}}{\left( \sqrt{v+(z-\mu)^{'}\gamma^{-1}(z-\mu)\zeta^{'}\gamma^{-1}\zeta} \right)^{-1-v/2}}, \\ & \mu=-\frac{v}{v-2}\zeta, \\ & \gamma=\frac{v-2}{v}\left( I_{2}-\frac{2v^{2}}{(v-2)^{2}(v-4)}\zeta\zeta^{'} \right), \\ & C(c,\gamma)=\frac{2^{-v/2}}{\Gamma(v/2)(v\pi)|\gamma|^{1/2}}, \end{matrix}$$

where $K$ is the modified Type III Bessel function, and $\zeta$ is an asymmetric parameter value of $(0.2,0.2)^{'}$ the vector of $v=5$ ,and $I_{2}$ is the unit matrix of $2\times2$.

In the setting of the above data generation process, the original hypothesis is the commonly used DCC-GJRGARCH model, and the standardized error term $\varepsilon_{jit}=\left( \varepsilon_{j,t},\varepsilon_{i,t} \right)^{'}$ is subject to a binary student-$t$ distribution. Only the conditional mean of $A_{1}$ is not set correctly, while the others are set correctly. Only the conditional variances of $A_{2}$ and $A_{3}$ are not correctly set, and only the conditional correlation of $A_{4}$ and $A_{5}$ was not set correctly, and the standardized error terms $z_{jit}$ of $A_{6}$ and $A_{7}$ are not set correctly.

For Monte Carlo simulations, we estimated the above model parameters using conditional maximum likelihood estimation (CMLE) and in-sample data, and used out-of-sample data to compute $u_{it}、u_{jit}$ and test statistics ${IND}_{CoES}(m)$, $m=1$ and $3. {IND}_{CoES}(m)$ converge to $N(0,1)$ and $\chi_{m}^{2}$ limiting distributions respectively. According to the above theoretical derivation process, these limiting distributions are appropriate only when $n/T$ are small. In addition, we choose the confidence level $\alpha$ = $\beta$ = 0.1, the reason why we do not choose a smaller confidence level is to consider that the joint violation sequences $h_{t}=1$ needs to have sufficient observations to ensure the asymptotic theory is valid. Finally, we set the in-sample observation sizes to $T=1000$ and 2500, the out-of-sample observation size $n=500$ and perform 1000 simulations.

The results of the Monte Carlo simulations are reported in Table A-1, where Panel A sets $T=1000$ and $n=500$ , Panel B sets $T=2500$ and $n=500$. First, it can be seen that the conditional coverage test ${IND}_{COES}(m)$ size are approximately equal to the $5\%$ level of significance, and the size distortion of the test statistic depends on the size of the $n/T$. The smaller the value of $n/T$, the lower the size distortion. Second, the conditional coverage test ${IND}_{CoES}(m)$ has strong statistical power against alternative $A_{6}$, and the conditional coverage test ${IND}_{CoES}(m)$ also has statistical power against alternatives $A_{1}-A_{3}$ and $A_{7}$. Overall, the Monte Carlo simulation results confirm that the conditional coverage test ${IND}_{CoES}(m)$ in this paper have strong statistical power.

**Table A-1**

Monte Carlo simulation results at 5% Significance Level

|  | $H_{0}$ | $A_{1}$ | $A_{2}$ | $A_{3}$ | $A_{4}$ | $A_{5}$ | $A_{6}$ | $A_{7}$ |
| --- | --- | --- | --- | --- | --- | --- | --- | --- |
|  | Panel A. $T=1000,n=500$ , Size and Power $(n/T=1/2)$ | | | | | | |  |
| ${IND}_{CoES}(1)$ | 0.059 | 0.179 | 0.199 | 0.026 | 0.065 | 0.064 | 0.531 | 0.172 |
| ${IND}_{CoES}(3)$ | 0.087 | 0.155 | 0.265 | 0.130 | 0.090 | 0.093 | 0.550 | 0.202 |
|  | Panel B. $T=2500,n=500$ , Size and Power $(n/T=1/5)$ | | | | | | |  |
| ${IND}_{CoES}(1)$ | 0.045 | 0.150 | 0.166 | 0.041 | 0.055 | 0.046 | 0.519 | 0.189 |
| ${IND}_{CoES}(3)$ | 0.073 | 0.128 | 0.232 | 0.137 | 0.092 | 0.074 | 0.546 | 0.216 |

**Appendix D. Daily-Frequency Tail-Risk Network Construction and Validation**

This appendix presents the results of reconstructing our tail-risk network at a daily frequency, as a robustness check addressing the regulatory relevance of our methodology. Using the same parametric and nonparametric tail-dependence estimation pipeline and network construction procedures described in the main text, we derive daily-frequency systemic risk indicators for the full sample of 55 financial institutions. The results below serve two purposes: (i) to verify whether the core network topology is robust across frequencies, and (ii) to assess the impact of microstructure noise inherent in daily equity returns on the stability and informativeness of the indicators.

D.1. Conditional Coverage Test Results

Table A-2 reports the average conditional coverage test p-values and rejection rates for the CoES daily tail-risk network over the period from July 2011 to June 2017. Table A-3 presents the corresponding results for the period from July 2017 to June 2023.

**Table A-2**

Average backtest p-values and rejection rates for the CoES daily tail-risk network (July 2011 – June 2017).

|  |  | July 2011 – June 2013 | | July 2013 – June 2015 | | July 2015 – June 2017 | |
| --- | --- | --- | --- | --- | --- | --- | --- |
| 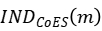 | | 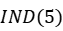 | 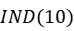 | 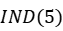 | 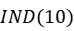 | 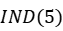 | 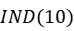 |
| Real Estate | P value | 0.9661 | 0.8707 | 0.8003 | 0.8069 | 0.6880 | 0.7447 |
|  | Rejection rate | 1.62% | 8.56% | 14.81% | 14.12% | 22.57% | 20.72% |
| Bank | P value | 0.8102 | 0.7438 | 0.8353 | 0.8364 | 0.7035 | 0.7343 |
|  | Rejection rate | 15.74% | 20.14% | 14.00% | 13.89% | 22.92% | 20.95% |
| Securities | P value | 0.9737 | 0.6675 | 0.8613 | 0.8631 | 0.7047 | 0.7476 |
|  | Rejection rate | 1.85% | 28.55% | 10.03% | 10.34% | 23.30% | 19.14% |
| Insurance | P value | 0.9804 | 0.8322 | 0.7596 | 0.7783 | 0.6761 | 0.7334 |
|  | Rejection rate | 0.46% | 12.04% | 17.59% | 15.28% | 23.61% | 18.98% |
| Others | P value | 0.9342 | 0.7173 | 0.8954 | 0.8862 | 0.6866 | 0.7156 |
|  | Rejection rate | 3.17% | 19.31% | 8.73% | 9.79% | 24.34% | 22.22% |
| Overall | P value | 0.9194 | 0.7671 | 0.8329 | 0.8358 | 0.6951 | 0.7378 |
|  | Rejection rate | 5.89% | 17.91% | 12.96% | 12.76% | 23.13% | 20.51% |

Note: IND(m) is the m-th order statistic for conditional coverage test. CoES’s confidence level α = β = 0.05. For reporting convenience, the report industry-level averages for p-value and rejection rate are reported.

**Table A-3**

Average backtest p-values and rejection rates for the CoES daily tail-risk network (July 2017 – June 2023).

|  |  | July 2017 – June 2019 | | July 2019- June 2021 | | July 2021 – June 2023 | |
| --- | --- | --- | --- | --- | --- | --- | --- |
| 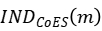 | | 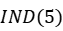 | 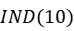 | 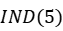 | 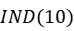 | 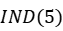 | 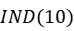 |
| Real Estate | P value | 0.8648 | 0.8393 | 0.8558 | 0.8769 | 0.8576 | 0.8671 |
|  | Rejection rate | 9.49% | 11.00% | 11.34% | 9.84% | 11.00% | 10.07% |
| Bank | P value | 0.8135 | 0.8132 | 0.9445 | 0.9622 | 0.6924 | 0.6974 |
|  | Rejection rate | 15.05% | 15.05% | 3.24% | 2.20% | 28.24% | 28.13% |
| Securities | P value | 0.8527 | 0.8662 | 0.9620 | 0.9685 | 0.8691 | 0.8344 |
|  | Rejection rate | 9.57% | 9.10% | 2.78% | 1.54% | 10.65% | 14.35% |
| Insurance | P value | 0.9033 | 0.8987 | 0.9771 | 0.9851 | 0.8805 | 0.8822 |
|  | Rejection rate | 6.02% | 7.41% | 1.39% | 0.93% | 9.72% | 10.65% |
| Others | P value | 0.7331 | 0.7892 | 0.6138 | 0.6946 | 0.8630 | 0.8338 |
|  | Rejection rate | 19.05% | 13.76% | 28.04% | 20.63% | 11.64% | 13.23% |
| Overall | P value | 0.8333 | 0.8355 | 0.8828 | 0.9063 | 0.8144 | 0.8075 |
|  | Rejection rate | 12.09% | 11.85% | 8.52% | 6.53% | 15.93% | 16.70% |

Note: IND(m) is the m-th order statistic for conditional coverage test. CoES’s confidence level α = β = 0.05. For reporting convenience, the report industry-level averages for p-value and rejection rate are reported.

Compared with the weekly-frequency results reported in the main text, the rejection rates in the IND(5) and IND(10) tests increase by approximately 5 to 15 percentage points in the daily-frequency analysis. The increase is most pronounced during the 2015–2016 Chinese stock market turbulence, a period characterized by extreme volatility and elevated levels of transient noise in daily trading. This pattern suggests that microstructure noise—such as bid-ask bounce, nonsynchronous trading, and short-lived liquidity-driven price dislocations—contaminates the tail-dependence estimates at the daily frequency, inflating the incidence of spurious tail connections and thereby elevating rejection rates in the coverage tests. These findings reinforce the methodological rationale for our use of weekly data in the main analysis: temporal aggregation attenuates noise and yields more stable and reliable tail-risk network estimates, particularly during stress episodes where noise amplification is most severe.

D.2. Topological Dynamics of the Daily-Frequency Network

Figures A-1 through A-4 display the time-series evolution of key topological characteristics of the daily-frequency tail-risk network from July 2011 to June 2023.


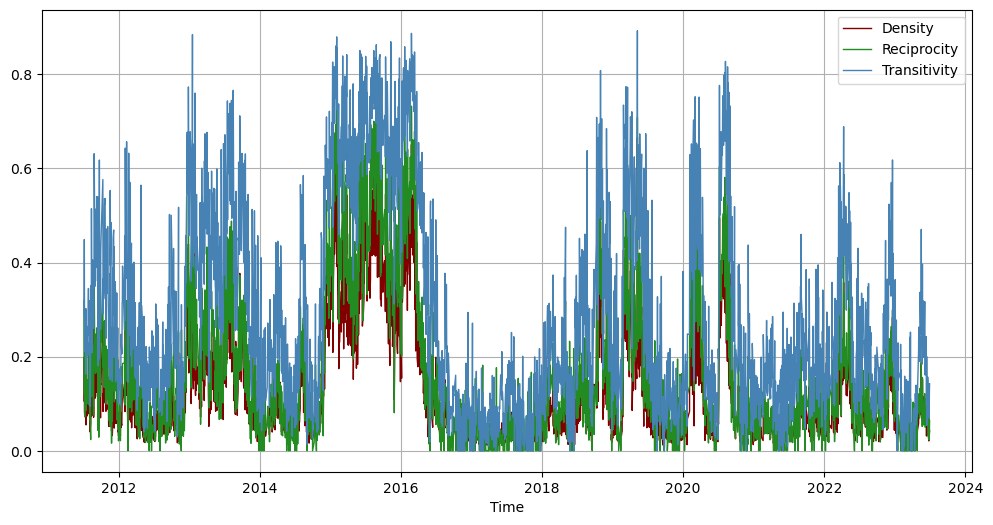
**Figure** A-1**.** Daily network density, reciprocity, and transitivity trends (July 2011 - June 2023).


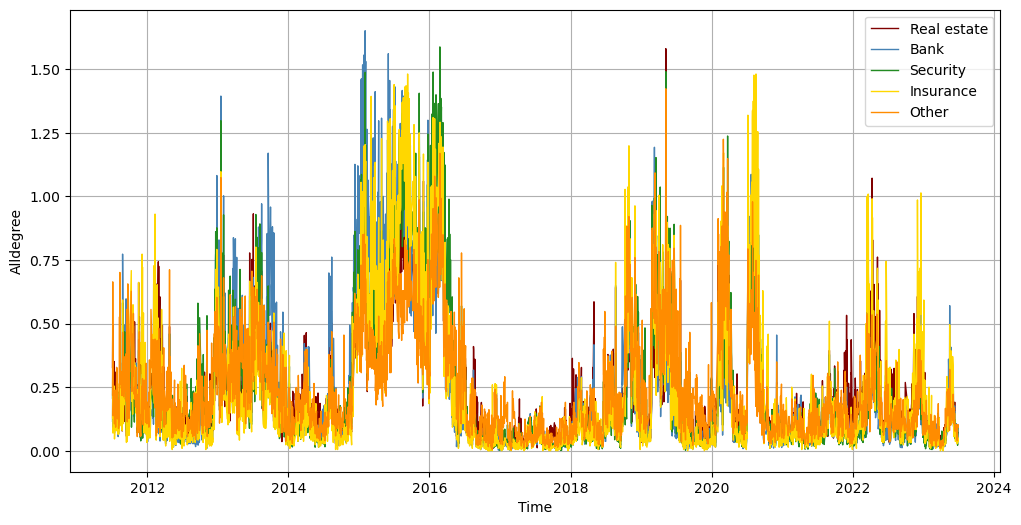
**Figure** A-2**.** Daily node all-degree trends for each sector (July 2011 - June 2023).


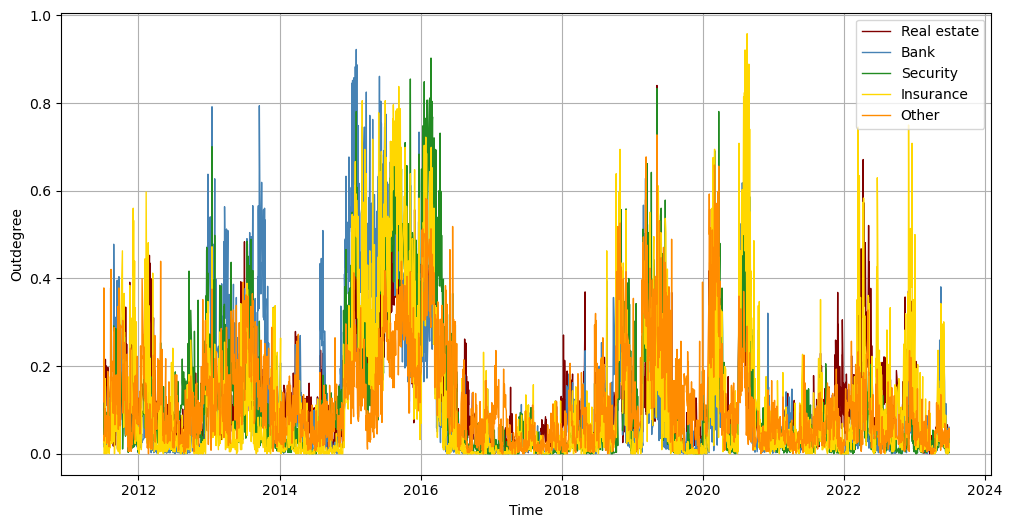
**Figure** A-3**.** Daily node out-degree trends in each sector (July 2011 - June 2023).


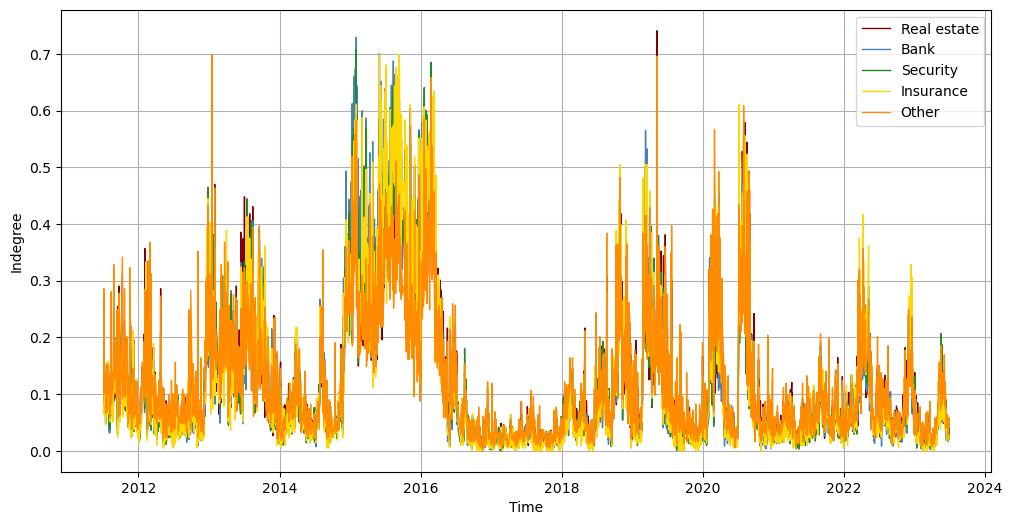
**Figure** A-4**.** Daily node in-degree trends in each sector (July 2011 - June 2023).

The daily-frequency topological indicators share broadly similar temporal trends with their weekly counterparts presented in the main text, confirming that the underlying systemic risk dynamics are consistently captured across frequencies. Network density, reciprocity, and transitivity all exhibit pronounced peaks during the 2015–2016 market turbulence and the 2020 COVID-19 shock, consistent with the main findings. However, the daily-frequency series display notably higher short-term volatility and reduced signal-to-noise ratios, which diminish the visual interpretability and informational clarity of the topological trends. This reduced readability is a direct consequence of the microstructure noise embedded in daily returns, which propagates through the tail-dependence estimation and into the network topology.

Taken together, the results in this appendix support two conclusions. First, the tail-risk network methodology is frequency-robust: the same systemic risk architecture is identifiable at both daily and weekly frequencies, and the key crisis episodes are consistently detected. This addresses the regulatory relevance concern by demonstrating that the network indicators can, in principle, be constructed at the daily cadence required for operational monitoring. Second, the superior signal-to-noise properties of weekly aggregation validate our methodological choice for the main analysis, where the objective is to identify the structural drivers of systemic risk through regression analysis—a task for which the noise reduction afforded by temporal aggregation is essential.
