# Supplementary material for: Dynamic financial tail risk networks: A backtesting-based conditional expected shortfall approach
Source: PLoS One. 2026 Jun 24;21(6):e0351966. doi: 10.1371/journal.pone.0351966 (PMC13293451; doi:10.1371/journal.pone.0351966)
Supplement: Attachment — Submitted filename: Review.pdf [file pone.0351966.s003.pdf]

Review of "Dynamic Financial Tail Risk Networks: A Backtesting-based Conditional Expected Shortfall Approach"

Summary

This paper develops a Factor-Copula methodology for constructing high-dimensional dynamic tail risk networks based on Conditional Expected Shortfall (CoES), introduces a comprehensive backtesting framework, and applies the methodology to 55 Chinese financial institutions. While the paper makes several contributions to systemic risk measurement, substantial revisions are required before publication.

Major Strengths

1. The paper addresses a genuine limitation in existing literature by combining Factor Copula models with tail risk network construction, overcoming the curse of dimensionality.

2. The development of a backtesting framework for CoES is novel and potentially valuable for practitioners.

3. The empirical application provides interesting insights into China's financial system across three major crisis periods.

4. The finding that leverage and local government debt behave differently than theoretical predictions in the Chinese context is intriguing.

Major Concerns Requiring Revision

1. Mathematical Framework and Exposition

- **Issue**: The transition from equations (7)-(10) describing the Factor Copula model is abrupt and lacks intuition. The GAS dynamic process for factor loadings (λ_{it}) needs clearer explanation.

- **Required revision**: Provide step-by-step derivation of how the Factor Copula captures dependencies. Explain why GAS dynamics are chosen over alternatives. Add a subsection explaining the economic intuition behind the model structure.

- **Issue**: The notation switches between different indexing conventions (sometimes $i|j$, sometimes i, j, sometimes ij).

- **Required revision**: Standardize notation throughout. Provide a notation table at the beginning.

2. Methodological Justification

- **Critical Issue**: The threshold method (Equation 20) using 75th percentile + 2*IQR appears arbitrary. Why this specific formula? How sensitive are results to this choice?

- **Required revision**: Provide theoretical or empirical justification for the threshold. Include robustness checks with alternative thresholds (e.g., 70th, 80th percentiles, 1.5*IQR, 2.5*IQR). Show how network topology changes with different thresholds.

- **Issue**: The simulation approach uses 10,000 iterations. Is this sufficient for stable estimates?

- **Required revision**: Provide convergence diagnostics. Show that key statistics stabilize with this number of simulations.

3. Backtesting Framework

- **Issue**: The cumulative joint violation process (Equation 13) derivation is unclear. The connection to Fubini's theorem needs elaboration.

- **Required revision**: Provide detailed proof in the main text (not just appendix). Explain why this approach is superior to alternatives. Include simulation evidence showing the test has correct size and good power properties.

- **Issue**: Tables 2-3 show rejection rates but don't discuss what constitutes acceptable performance.

- **Required revision**: Add benchmarks or significance tests. What rejection rate would indicate model failure?

4. Empirical Analysis

- **Major Issue**: The network visualizations (Figure 5) are nearly impossible to interpret. The dense overlapping edges provide little insight.

- **Required revision**: Use alternative visualization methods (e.g., heatmaps, hierarchical layouts, or separate plots for different edge strength categories). Consider interactive visualizations in supplementary materials.

- **Issue**: Section 4 on determinants seems disconnected from the network analysis. How do the regression results relate to the network topology findings?

- **Required revision**: Explicitly link network centrality measures to the regression analysis. Show how institutions identified as systemic in the network relate to the size/leverage findings.

- **Issue**: The explanation for negative leverage effect and local government debt impact is speculative.

- **Required revision**: Provide additional empirical evidence. Consider subsample analysis (SOEs vs private, banks vs non-banks) to test the proposed mechanisms.

5. Robustness and Validation

- **Critical Gap**: No robustness checks for key modeling choices.
- **Required revision**: Add section with:
  - Alternative copula specifications (e.g., vine copulas)
  - Different numbers of factors in the Factor Copula
  - Alternative risk measures (MES, SRISK)
  - Subsample stability (pre/post 2015 reforms)
  - Out-of-sample validation
- **Issue**: No comparison with simpler benchmark models.
- **Required revision**: Compare network properties with those from DCC-GARCH or simple correlation-based networks.

6. Literature Review and Positioning

- **Issue**: Limited discussion of recent Factor Copula developments and alternative high-dimensional dependence models.
- **Required revision**: Expand literature review to include recent work on vine copulas, factor models in systemic risk, and alternative network construction methods. Clearly position contributions relative to Oh and Patton (2018).

Minor Issues

1. Abstract should quantify main findings (e.g., "reduces rejection rates by X%").
2. Introduction is too long. Move some technical details to methodology section.
3. Tables 4-6 need statistical significance indicators for rankings.
4. Figure labels are too small to read.
5. Equation (10) has inconsistent notation (ω_{2i} appears without definition).
6. The conclusion is too brief and doesn't discuss limitations adequately.
7. Data and code availability statement is vague. Provide specific repository links.

Specific Technical Comments

1. Page 10, Equation (10): The notation $s_{i,t}$ is used before being defined.
2. Page 12, Line 5: "edge-wise backtesting" needs formal definition.
3. Page 16: Why use weekly returns rather than daily? Justification needed given the importance of capturing tail events.
4. Table 1: Consider grouping institutions more clearly by sector.
5. Page 20: The claim that network metrics "align with real market fluctuations" needs quantitative support (correlation analysis).

Recommendations

This paper makes potentially important contributions but requires major revisions:

1. **Essential**: Add comprehensive robustness checks and improve mathematical exposition.
2. **Essential**: Provide better justification for methodological choices (thresholds, simulations).
3. **Essential**: Improve visualization and interpretation of network results.
4. **Important**: Strengthen the connection between network analysis and regression results.
5. **Important**: Expand empirical analysis to better support the leverage and government debt findings.
6. **Suggested**: Consider splitting into two papers - one on methodology, one on empirical application.

The paper should be reconsidered after major revisions addressing these concerns. The authors should particularly focus on robustness, clearer exposition, and better integration of the different analytical components.
