# Supplementary material for: Dynamic financial tail risk networks: A backtesting-based conditional expected shortfall approach
Source: PLoS One. 2026 Jun 24;21(6):e0351966. doi: 10.1371/journal.pone.0351966 (PMC13293451; doi:10.1371/journal.pone.0351966)
Supplement: Attachment — Submitted filename: Referee_Report_PONE-D-25-35082.pdf [file pone.0351966.s004.pdf]

Referee Report for PONE-D-25-35082

This paper develops a Factor-Copula methodology for constructing high-dimensional dynamic tail risk networks based on the conditional expected shortfall (CoES) to overcome the limitations of traditional quantile regression and copula models. The authors backtest the CoES based on cumulative joint violations and conditional coverage tests. The dynamic analysis indicates that the network topology characteristics align with real market fluctuations, while quantitative analyses show different effects of institutional and market factors on network-measured risk spillovers and contagion among Chinese financial institutions.

This is an interesting paper, and my comments for further improvement are as follows:

1. There is currently a vast and growing academic literature on this topic, and it is unclear how this paper adds and differentiates from other studies on copula modelling. The authors should make much more effort to explain their contributions both in the Introduction as well as throughout the paper where necessary.

2. The proposed framework is based on several subjective assumptions around the various aspects of the model. The authors should carefully discuss the relevant issues and justify these choices more thoroughly in their paper.

3. Copula models have profound implications on portfolio management and optimization. I would have liked to see a new section in the paper where the authors will apply their proposed copula framework within a portfolio context with real data and highlight the added economic value of their approach.

4. Given point (3), the authors should also cite and discuss several papers related to portfolio management and optimization under parameter uncertainty both in the introduction as well as throughout the paper, see for example: Board and Sutcliffe (1994, MS), Platanakis and Sutcliffe (2017, EJF), Platanakis, Sutcliffe and Ye (2021, EJOR), Huang et al. (forthcoming, IJF), Kan, Wang and Zhou (2022, MS), Oikonomou, et al. (2018, BAR), Platanakis and Urquhart (2019, EL), Yuan and Zhou (2024, JFQA), and Lassance, et al. (2024, MS), among others.

5. The author(s) should discuss somewhere in the paper how their proposed framework could benefit from the incorporation of machine learning (ML) techniques, see Doumpos, et al. (2023, EJOR). For example, the authors could check carefully the ML methods described in Doumpos, et al. (2023, EJOR) and discuss how such ML methods can enhance further their method.

6. The design of the various tables and figures in the paper needs serious improvement. Further, the author(s) should conduct a serious proof-reading to correct the various typos throughout the entire paper.

7. Finally, the references are not in alphabetical order, and this needs to be corrected.

References

Board, J. L., & Sutcliffe, C. M. (1994). Estimation methods in portfolio selection and the effectiveness of short sales restrictions: UK evidence. *Management science*, *40*(4), 516-534.

Doumpos, M., Zopounidis, C., Gounopoulos, D., Platanakis, E., & Zhang, W. (2023). Operational research and artificial intelligence methods in banking. *European Journal of Operational Research*, *306*(1), 1-16.

Huang, X., Newton, D. P., Platanakis, E., & Sutcliffe, C. (2024). Single-stage portfolio optimization with automated machine learning for M6. *International Journal of Forecasting*.

Kan, R., Wang, X., & Zhou, G. (2022). Optimal portfolio choice with estimation risk: No risk-free asset case. *Management Science*, *68*(3), 2047-2068.

Lassance, N., Martin-Utrera, A., & Simaan, M. (2024). The risk of expected utility under parameter uncertainty. *Management Science*, *70*(11), 7644-7663.

Oikonomou, I., Platanakis, E., & Sutcliffe, C. (2018). Socially responsible investment portfolios: does the optimization process matter? *The British Accounting Review*, *50*(4), 379-401.

Platanakis, E., & Sutcliffe, C. (2017). Asset–liability modelling and pension schemes: the application of robust optimization to USS. *The European Journal of Finance*, *23*(4), 324-352.

Platanakis, E., & Urquhart, A. (2019). Portfolio management with cryptocurrencies: The role of estimation risk. *Economics Letters*, *177*, 76-80.

Platanakis, E., Sutcliffe, C., & Ye, X. (2021). Horses for courses: Mean-variance for asset allocation and 1/N for stock selection. *European Journal of Operational Research*, *288*(1), 302-317.
