# Supplementary material for: Dynamic financial tail risk networks: A backtesting-based conditional expected shortfall approach
Source: PLoS One. 2026 Jun 24;21(6):e0351966. doi: 10.1371/journal.pone.0351966 (PMC13293451; doi:10.1371/journal.pone.0351966)
Supplement: Attachment — Submitted filename: Response to Reviewers .docx [file pone.0351966.s007.docx]

Dear Editors and Reviewers:

Thank you for your letter and for the reviewers’ comments concerning our manuscript entitled “**Dynamic Financial Tail Risk Networks: A Backtesting-based Conditional Expected Shortfall Approach**” (**ID: PONE-D-25-35082**). Those comments are all valuable and very helpful for revising and improving our paper, as well as the important guiding significance to our research. We have studied comments carefully and have made corrections which we hope meet with approval.

Reviewer #1

1. The transition from equations (7)-(10) describing the Factor Copula model is abrupt and lacks intuition. The GAS dynamic process for factor loadings ($\lambda_{i,t}$) needs clearer explanation.

***The author‘s answer:*** Based on the reviewers' comments, we have expanded the explanations of marginal distribution modeling and factor copula modeling, ensuring that each symbol and variable in the formulas is sufficiently explained. In lines 175–189, we provide a detailed exposition of the marginal distribution modeling. In lines 190–224, we elaborate on the factor copula and the GAS model. In particular, between lines 202 and 206, we clarify the advantages of the GAS framework and explain why it is well‑suited for the factor copula specification adopted in this study.

2. The notation switches between different indexing conventions (sometimes i|j, sometimes i, j, sometimes ij).

***The author‘s answer:*** In response to the reviewers’ comments, we have unified the notation of subscripts throughout the manuscript and ensured that all symbols and variables are explained in the immediate context. Specifically, the subscripts for entity i and time t are now consistently separated by a comma, e.g., i,t. Furthermore, entity i conditional on entity j is uniformly denoted as i|j.

3. The threshold method (Equation 20) using 75th percentile+ 2*IQR appears arbitrary. Why this specific formula? How sensitive are results to this choice?
 ***The author‘s answer:*** Based on the reviewers' feedback, we have revised the method for selecting the threshold of the tail‑risk network. Specifically, the threshold is now determined using the knee‑point detection method applied to the global efficiency curve. In lines 290–308, we have added an explanation of the definition of global efficiency, described the advantages of the knee‑point detection approach for threshold selection, and highlighted the merits of global efficiency relative to information entropy and network density. Correspondingly, lines 413–426 present the resulting threshold value.

Figure 2 shows how network global efficiency, information entropy, and network density vary as the percentile threshold increases. We provide a detailed interpretation of the patterns observed in the figure and explain why the threshold identified by the knee point of global efficiency is adopted. Owing to this change in threshold, all subsequent analyses (except for the backtesting analysis) have been updated accordingly. The results, however, remain largely unchanged, indicating that the risk network constructed in this study is not sensitive to the choice of threshold.

4. The simulation approach uses 10,000 iterations. Is this sufficient forstable estimates?
 ***The author‘s answer:*** Following the reviewers' suggestions, we have added convergence diagnostics for the tail dependence coefficient (TDC) and tail correlation (TC) in lines 350–362, along with brief explanations of these two metrics. In Figure 1, we present the convergence diagnostics for four randomly selected institutional pairs from a trading week, all of which show clear convergence. The convergence patterns observed for other sampled pairs are similar.

5. The cumulative joint violation process (Equation 13) derivation is unclear. The connection to Fubini’s theorem needs elaboration.

***The author‘s answer:*** In response to the reviewers' suggestion, we have added a detailed derivation of the expectation of $H_{t}\left( \alpha,\beta,\theta\right)$ in Equation 15 based on Fubini's theorem. Moreover, to help readers better understand Equation 16, we added the probability expression for $h_{t}\left( \alpha,\beta,\theta\right)$ in the first line, which can be derived from the definition of the CoVaR.

Following the reviewers' suggestion, we considered moving the size and power tests of the backtesting metrics from the Appendix to the main text. This adjustment, however, raises two concerns. First, the appendix material is based on conventional out-of-sample forecasting, while the main text focuses exclusively on in-sample analysis—a necessary approach given the unobservable nature of systemic risk. Introducing out-of-sample content could therefore confuse readers. Second, relocating this material would disrupt the paper’s thematic coherence and structure. Instead, we have enriched the main text with a more detailed mathematical derivation of the backtesting metrics. This addition clarifies the rationale behind the diagnostics and strengthens the justification for using them to validate network estimation accuracy.

6. Tables 2-3 show rejection rates but don’t discuss what constitutes acceptable performance.
 ***The author‘s answer:*** As few previous studies have used backtesting rejection rates to assess the performance of network-construction models, it is difficult to establish a reasonable rejection-rate threshold based on prior experience. Following the reviewers' suggestion, we instead evaluate whether the difference in rejection rates between two models is statistically significant, thereby determining whether the performance difference between the network-construction models is significant. We accomplish this via the McNemar test, and in lines 375-390 we have added an explanation of the rationale for using this test and an analysis of its results. Table 4 presents the results of the McNemar tests, which complement the rejection-rate comparisons in Tables 2 and 3 by statistically demonstrating that the rejection-rate advantage of the Factor-Copula-CoES model over the benchmark models is significant.

7. The network visualizations (Figure 5) are nearly impossible to interpret. The dense overlapping edges provide little insight.

***The author‘s answer:*** In response to the reviewers' comments, we have split the original figure into four separate, clearer heatmaps, as shown in Figures 7–10. In lines 462–469, we provide an analysis and interpretation of the content of these four figures. The new heatmaps highlight the industries to which the nodes belong, enabling a clearer visualization of which financial sectors exhibit concentrated risk.

8. Section 4 on determinants seems disconnected from the network analysis. How do the regression results relate to the network topology findings?
 ***The author‘s answer:*** Following the reviewer's suggestion, we have added a transitional paragraph in the first part of Section 4 (in lines 495-498) to better connect the network topology analysis with the investigation of tail risk determinants:’ The topological analysis of the tail‑risk network offers a valuable perspective for risk decomposition, enabling us to distinguish between two distinct channels of systemic risk: risk spillover and risk contagion. Unlike conventional systemic‑risk studies that rely on CoVaR (or CoES) metrics, this framework allows for a clear separation of spillover and contagion effects.’

Following the reviewers' suggestion, we attempted to report the assets and liabilities of the systemically important institutions identified in Tables 5–7 in tabular form. However, the resulting table does not demonstrate that institutions with higher assets or liabilities are more likely to be identified as systemically important. This outcome is quite reasonable, as there are multiple determinants of systemic risk, and assets and liabilities are only one of them. This also underscores the necessity of conducting regression analysis for quantitative investigation. While the topological network analysis focuses on examining the time‑varying characteristics of network‑topology metrics, the regression‑based quantitative analysis aims to uncover the determinants driving the temporal dynamics of these network‑topology indicators.

9. The explanation for negative leverage effect and local government debt impact is speculative.
 ***The author‘s answer:*** In line with the reviewer's suggestion, we have removed the speculative content regarding the negative leverage effect and the impact of local government debt, and now only report the regression results objectively in the main text. As a compensatory measure, we have moved that discussion to the "Future Research" section in the conclusion, as shown in lines 595–608. Conducting additional empirical research on leverage and local government debt could shift the focus of the paper toward studying the specificities of China’s financial markets. We prefer to pursue that line of inquiry in future work.

10. No robustness checks for key modeling choices.
 ***The author‘s answer:*** In summary, this paper proposes six tail‑risk network construction models: static‑copula‑CoVaR, time‑varying‑copula‑CoVaR, factor‑copula‑CoVaR, static‑copula‑CoES, time‑varying‑copula‑CoES, and factor‑copula‑CoES. Model robustness is evaluated through backtesting, and on the basis of the backtest rejection‑rate metric, the factor‑copula‑CoES model is selected as the preferred specification.

During the benchmark‑model selection stage, we considered including a Vine‑copula model, but found it unsuitable for constructing high‑dimensional dynamic tail‑risk networks in the present context. Vine‑copula models face a severe “curse of dimensionality” when applied to high‑dimensional data: for an m‑dimensional variable set, they require m‑1 structure trees and m(m‑1)/2 edges (bivariate copulas), each of which introduces parameters to be estimated. The number of parameters grows quadratically with dimension, leading to prohibitive computational costs, a problem that becomes even more acute when time‑varying copulas are introduced.

The factor‑copula model employed in this paper—which incorporates one common factor and one idiosyncratic factor—is sufficiently flexible to approximate any copula with a closed‑form density, and maintains a favorable balance between computational resources and runtime when simulating copulas without closed‑form densities. Adding further factors could disrupt this balance.

Although both MES (or SRISK) and CoVaR (or CoES) are widely used tail‑risk measures, the tail risks they capture are not equivalent. MES is computed from the tail distribution of system returns and the mean (unconditional) distribution of individual returns, whereas CoVaR relies on the joint tail distribution of individual and system returns. In the context of tail‑risk network construction, the resulting network is asymmetric and directed. This requires that the edge relationship between nodes i and j be defined in a mutually symmetric way, so that any observed asymmetry in the network can be attributed to genuine risk‑spillover patterns rather than to modeling artifacts. The CoVaR measure satisfies this requirement, because both directions of the edge—CoVaR(i|j) and CoVaR(j|i)—can be interpreted as a tail‑risk‑to‑tail‑risk influence.

Given the unobservable nature of systemic risk, in‑sample analysis constitutes an essential component of systemic‑risk research and is a primary focus of this paper. Our aim is to use the factor‑copula framework and the CoES metric to adequately capture the dependence among financial institutions, thereby examining the historical evolution of systemic risk—which also explains why the paper does not engage in out‑of‑sample forecasting.

11. No comparison with simpler benchmark models.

***The author‘s answer:*** Given that the research focus of this paper is the construction of high‑dimensional dynamic complex networks, both DCC‑GARCH and quantile‑regression models have shown limitations in performance within this context. We experimented with the DCC‑GARCH model to capture the time‑varying correlations among the 55 financial institutions, but found that it performed poorly in tracking the time‑varying nature of sample correlations. Therefore, we decided to set the benchmark models within the copula‑based framework, which also facilitates a more coherent comparison across model specifications.

12. Limited discussion of recent Factor Copula developments and alternative high-dimensional dependence models.

***The author‘s answer:*** Following the reviewer's suggestion, we have added a summary of recent developments in factor copula models in lines 63–71. However, the current literature still lacks representative studies that explicitly apply factor copula models to the construction of financial risk networks, which further underscores the academic significance of the present work.

13. Abstract should quantify main findings (e.g., “reduces rejection rates by X%”).

***The author‘s answer:*** Following the reviewers' suggestion, we have added the following sentence as the third sentence of the abstract: ‘The proposed factor‑copula‑CoES model reduces the rejection rates in 10‑th order conditional backtests by 56.37%, 47.72%, and 1.96% relative to the static‑copula‑CoES, time‑varying‑copula‑CoES, and factor‑copula‑CoVaR models, respectively, with all reductions being statistically significant.’

14. Introduction is too long. Move some technical details to methodology section.

***The author‘s answer:*** Following the reviewers' suggestions, we have shortened the introduction by approximately 150 words. The deleted content includes repeated explanations of the paper's innovations and speculative remarks about the regression results concerning liabilities and the dgo variable.

15. Tables 4-6 need statistical significance indicators for rankings.

***The author‘s answer:*** Thank you for the suggestion. The rankings presented in Tables 5–7 are based on descriptive network centrality metrics (out‑degree and in‑degree) derived from the tail‑risk adjacency matrices. Since these rankings reflect deterministic structural properties of the observed network—not estimates subject to sampling variability—statistical significance indicators are not applicable here. Such centrality-based rankings are commonly reported in the network literature as descriptive measures of node-level systemic importance.

16. Figure labels are too small to read.

***The author‘s answer:*** Following the reviewers' suggestion, we have increased the font size of the labels in the figures throughout the paper.

17. Equation (10) has inconsistent notation (ω2i appears without definition).

***The author‘s answer:*** Following the reviewers' suggestions, we have revised all formulas in that section to ensure that every newly introduced symbol is explained subsequently in the text.

18. The conclusion is too brief and doesn’t discuss limitations adequately

***The author‘s answer:*** Following the reviewers' suggestion, we have added content on future research directions in lines 595–624, introducing three potential avenues for further investigation. We have also moved the speculative remarks on leverage and dgo variables from the tail‑risk determinants analysis to the future research section, where a well‑designed mediation‑effect model could be employed to verify these anomalies.

19. Data and code availability statement is vague. Provide specific repository links.

***The author‘s answer:*** The file we provided integrates four folders—Copula, Data, Network, and Regression—containing all the code and data used in this paper. During the subsequent publication process, we will upload the files to the required repository as specified by the journal and provide the corresponding link.

20. Page 10, Equation (10): The notation si,t is used before being defined.

***The author‘s answer:*** Following the reviewers' suggestions, we have revised all formulas in that section to ensure that every newly introduced symbol is explained subsequently in the text.

21. Page 12, Line 5: “edge-wise backtesting” needs formal definition.

***The author‘s answer:*** Following the reviewers' suggestions, we have removed the expression "edge-wise" and revised it as follows (in lines 80):’ Thus, by backtesting each edge in a CoVaR/CoES-defined tail-risk network, we ensure the reliability of the network as a whole.’

22. Why use weekly returns rather than daily? Justification needed given the importance of capturing tail events.
 ***The author‘s answer:*** Weekly returns effectively filter out high‑frequency noise present in daily data, thereby providing a more stable and reliable signal for detecting and quantifying systemic tail‑risk dependencies.

23. Table 1: Consider grouping institutions more clearly by sector.

***The author‘s answer:*** We have added borders to the tables, grouping institutions belonging to the same industry within shared frames.

24. Page 20: The claim that network metrics “align with real market fluctuations” needs quantitative support (correlation analysis).

***The author‘s answer:*** Following the reviewers' suggestions, we have revised the corresponding sentence in the abstract to: "The dynamic analysis of the tail risk network of Chinese listed financial institutions reveals that its topological characteristics align closely with real‑world market risk events." This revision more accurately conveys the rationale underlying the network‑topology findings.

Reviewer #2

1. There is currently a vast and growing academic literature on this topic, and it is unclear how this paper adds and differentiates from other studies on copula modelling. The authors should make much more effort to explain their contributions both in the Introduction as well as throughout the paper where necessary

***The author‘s answer:*** In the introduction section, lines 63–71, we have added a summary of recent advances in factor copula models. Very few studies have applied factor copula to the construction of tail‑risk networks; this paper aims to fill that gap. Moreover, few works have integrated backtesting with network construction, and we highlight this contribution in the literature review on backtesting.

2. The proposed framework is based on several subjective assumptions around the various aspects of the model. The authors should carefully discuss the relevant issues and justify these choices more thoroughly in their paper.
***The author‘s answer:*** Following the reviewers' suggestions, we have validated two key model parameters. First, we have changed the threshold for network construction from an empirical method to a global‑efficiency knee‑point detection approach. In lines 290–309, we have added an explanation of the knee‑point detection method and its advantages; in lines 413–425, we have also included an analysis of the threshold selection results, with Figure 2 presenting the corresponding analytical outcomes. Using the newly selected threshold, we have reconstructed the tail‑risk network, and all figures and tables in the paper—except those related to backtesting—have been updated accordingly. Nevertheless, the analytical conclusions remain consistent with the original findings. Second, we have examined the number of simulations in the factor‑copula model. In lines 350–362, we have added convergence diagnostics and analysis for the tail dependence coefficient and tail correlation, with Figure 1 showing the convergence results. The findings indicate that 10,000 simulation runs are sufficiently adequate.

3. Copula models have profound implications on portfolio management and optimization. I would have liked to see a new section in the paper where the authors will apply their proposed copula framework within a portfolio context with real data and highlight the added economic value of their approach.

***The author‘s answer:*** Since the focus of this paper is the identification and analysis of tail risk, we concentrate on in‑sample prediction. Given the unobservable nature of tail risk, in‑sample analysis is essential. Portfolio optimization is indeed an important application of copula models, but research in that area is generally designed around out‑of‑sample forecasting. Introducing portfolio‑optimization content here could confuse readers and blur the thematic focus of the paper.

4. Given point (3), the authors should also cite and discuss several papers related to portfolio management and optimization under parameter uncertainty both in the introduction as well as throughout the paper, see for example: Board and Sutcliffe (1994, MS), Platanakis and Sutcliffe (2017, EJF), Platanakis, Sutcliffe and Ye (2021, EJOR), Huang et al. (forthcoming, IJF), Kan, Wang and Zhou (2022, MS), Oikonomou, et al. (2018, BAR), Platanakis and Urquhart (2019, EL), Yuan and Zhou (2024, JFQA), and Lassance, et al. (2024, MS), among others.

***The author‘s answer:*** In the introduction section, lines 68–70, we have added a literature review covering recent applications of factor‑copula models to portfolio optimization:’ Chen et al. (2023) applied a dynamic factor copula‑based mean‑ES model to optimize portfolio risk in China’s real industry, showing the approach effectively captures time‑varying dependence in high‑dimensions.’

5. The author(s) should discuss somewhere in the paper how their proposed framework could benefit from the incorporation of machine learning (ML) techniques, see Doumpos, et al. (2023, EJOR). For example, the authors could check carefully the ML methods described in Doumpos, et al. (2023, EJOR) and discuss how such ML methods can enhance further their method.

***The author‘s answer:*** Following the reviewers' suggestions, we have added a research direction on the introduction of graph‑neural‑network artificial intelligence algorithms in the "Future Research" section (lines 616–625): ‘The dynamic tail‑risk network constructed here also creates opportunities for introducing graph‑based artificial intelligence algorithms such as graph neural networks (GNNs). In recent years, GNNs have advanced rapidly, owing to their strong ability to mine graph‑structured information and their high accuracy in classification and prediction tasks, and have been widely adopted in transportation, computer science, and social‑network analysis. However, their application in financial risk research faces two major challenges: correlation‑based models often suffer from the “curse of dimensionality,” and GNN‑type algorithms typically require large volumes of data. The high‑dimensional dynamic tail‑risk network proposed in this paper provides rich feature data for GNNs and allows them to fully utilize their network‑information mining capabilities. The integration of graph neural networks with tail‑risk networks shows considerable promise in financial domains such as systemic risk identification and prediction, as well as portfolio optimization.’.

6. The design of the various tables and figures in the paper needs serious improvement. Further, the author(s) should conduct a serious proof-reading to correct the various typos throughout the entire paper.

***The author‘s answer:*** Following the reviewers' suggestions, we have added borders to Table 1 to group institutions more clearly by industry; replaced the original tail‑risk network visualizations with heatmap representations, as shown in Figures 7–10; and increased the font size of all labels in the figures. Finally, we have proofread the entire manuscript and corrected any typos.

7. Finally, the references are not in alphabetical order, and this needs to be corrected.

***The author‘s answer:*** According to the journal's requirements, the references are now listed in the order of their first appearance in the main text. Since three new references have been added during this revision, the reference list has been updated accordingly.

Thank you very much for your attention and time. Look forward to hearing from you.

Yours sincerely.

Feng Shen, Professor

shenfeng@swufe.edu.cn
