# Supplementary material for: Dynamic financial tail risk networks: A backtesting-based conditional expected shortfall approach
Source: PLoS One. 2026 Jun 24;21(6):e0351966. doi: 10.1371/journal.pone.0351966 (PMC13293451; doi:10.1371/journal.pone.0351966)
Supplement: Attachment — Submitted filename: Response_to_Reviewers__auresp_2.docx [file pone.0351966.s008.docx]

Dear Editors and Reviewers:

Thank you for your letter and for the reviewers’ comments concerning our manuscript entitled “**Dynamic Financial Tail Risk Networks: A Backtesting-based Conditional Expected Shortfall Approach**” (**ID: PONE-D-25-35082R1**). Those comments are all valuable and very helpful for revising and improving our paper, as well as the important guiding significance to our research. We have studied comments carefully and have made corrections which we hope meet with approval.

Reviewer #1

**1. The paper uses weekly returns but regulatory frameworks (Basel III/FRTB, CCAR, market risk capital rules) explicitly require daily risk measures for compliance and capital adequacy.**

***Response to Reviewer#1 Comment 1:*** Daily Frequency for Regulatory Relevance

We sincerely thank the Reviewer for this incisive comment. We fully acknowledge that daily-frequency analysis is the operational standard for systemic risk surveillance in regulatory practice, as exemplified by the Basel Committee's frameworks for market risk monitoring and backtesting. We appreciate the Reviewer for compelling us to address this gap directly.

(1). Why weekly frequency is retained in the main empirical analysis

Our study follows a two-stage design: (i) constructing a tail-risk network to derive systemic risk indicators, and (ii) conducting regression analyses to identify their institutional and market-level factors drivers. The second-stage covariates are observed at monthly or quarterly frequencies. Upscaling these to daily frequency would require substantial interpolation, introducing artificial smoothness and inflating regression precision. Weekly aggregation thus represents a deliberate choice: it attenuates microstructure noise in daily equity returns while remaining the highest frequency at which meaningful alignment with macroeconomic covariates can be achieved. This approach is consistent with established practice in the systemic risk literature, where weekly or monthly network snapshots are employed when the analytical focus is on cross-sectional risk drivers rather than high-frequency early-warning signals.

(2). Daily-frequency network validation: New Appendix D

We agree that the above justification does not obviate the need for daily-frequency evidence. A regulator deploying our framework would reasonably ask whether the tail-risk network indicators are empirically robust at a daily frequency. We have therefore added a dedicated Appendix D to the manuscript containing the following:

Daily-frequency network reconstruction. We reconstructed the entire tail-risk network at a daily frequency using the same methodological pipeline applied to the same 55 institutions across banking, real estate, securities, and insurance.

Conditional coverage test results (Tables A-2 and A-3). We present average conditional coverage test p-values and rejection rates for two sub-periods (July 2011 – June 2017 and July 2017 – June 2023). Compared with the weekly-frequency results in the main text, rejection rates in the IND(5) and IND(10) tests increase by approximately 5 to 15 percentage points, with the largest increases occurring during the 2015–2016 stock market turbulence. This pattern indicates that microstructure noise in daily returns contaminates tail-dependence estimates, inflating spurious tail connections. These findings reinforce the methodological rationale for weekly aggregation in our main analysis.

Topological dynamics (Figures A-1 to A-4). We provide daily-frequency time-series plots of network density, reciprocity, transitivity, and sectoral degree centrality measures. The daily indicators share broadly similar temporal trends with their weekly counterparts, confirming that the underlying systemic risk architecture is consistently captured across frequencies. However, the daily series display notably higher short-term volatility and reduced signal-to-noise ratios, diminishing the visual interpretability of the topological trends relative to the weekly analysis.

Key conclusion from the appendix. The tail-risk network methodology is frequency-robust—the same systemic risk architecture is identifiable at both frequencies, and key crisis episodes are consistently detected. This addresses the regulatory relevance concern by demonstrating daily deployability. At the same time, the superior signal-to-noise properties of weekly aggregation validate our methodological choice for the main analysis, where the objective is to identify structural drivers through regression.

3. Adjustments to the main text

In the section 3.2 (line 404), we have inserted a passage that reads:” In addition, to assess whether our tail-risk network construction methodology is robust to the choice of data frequency, we replicate the entire analysis at a daily frequency. The results, detailed in Appendix D, confirm that the core network architecture is consistently captured across frequencies, while also revealing the effects of microstructure noise inherent in daily returns.” This addition explicitly cross-references the daily-frequency validation and signals to readers that the network methodology is frequency-agnostic.

**2. The paper fails to clearly specify its backtesting approach. Only from the authors’ response to reviewers can one determine that backtesting is performed in-sample across the full 2011-2022 period. By definition, backtesting requires out-of-sample evaluation with rolling estimation windows to avoid overfitting bias.**

***Response to Reviewer#1 Comment 2:*** Backtesting Methodology and Model Comparison Validity

We sincerely thank the Reviewer for this rigorous and important comment. We fully agree that out-of-sample evaluation with rolling estimation windows is the gold standard for studies whose primary contribution is forecasting or predictive model selection. The Reviewer's articulation of the four requirements—rolling/expanding windows, one-step-ahead forecasts, out-of-sample periods, and transparent re-estimation protocols—is entirely correct in that context.

We respectfully submit, however, that our study belongs to a different research paradigm, and that this distinction bears directly on the appropriate evaluation framework.

(1). Our study is measurement-oriented, not prediction-oriented

Our research question is: how can we measure and characterize systemic risk in the historical record using tail-risk networks? This is fundamentally a measurement problem. Systemic risk is not directly observable—it is a latent construct that must be inferred from observable co-movement patterns among financial institutions. The validity of our approach hinges not on forecasting future systemic events, but on whether the network indicators adequately capture the tail dependence structure that constitutes systemic risk in the historical data.

In this measurement paradigm, evaluating the model on the full historical sample is the primary analytical exercise, not overfitting. Just as one does not reserve a hold-out sample when estimating a factor model to describe the covariance structure of returns, or when fitting a copula to characterize tail dependence. In our works, tail risk networks are constructed and compared using the full available history, precisely because the goal is to describe the historical architecture of systemic risk rather than to generate out-of-sample forecasts.

(2). What our backtesting evaluates

The "backtesting" in our paper evaluates whether the estimated tail-risk network adequately captures the dependence structure among financial institutions during historical stress episodes. Specifically, we assess whether the tail-dependence estimates from our copula-based models satisfy conditional coverage properties—that is, whether the identified tail connections actually manifest as joint extreme movements in the data. This validates a measurement model on historical data, not a forecasting model on unseen data. Such in-sample validation of tail dependence structures is standard in the copula modeling literature, as the object of interest—the tail dependence parameter—is a feature of the joint distribution during the sample period, not a prediction about future observations.

(3). Recalibration of claims and scope

We acknowledge that our original manuscript may have used language that could be interpreted as making forecasting-oriented superiority claims. We will make the following revisions:

Reposition the study as measurement-oriented. We will revise the introduction and methodology sections to explicitly frame our contribution as the measurement and characterization of historical systemic risk architecture, clearly distinguishing this from forecasting.

Remove superiority language. We will replace all language suggesting "superiority" or "outperformance" with language describing the relative adequacy of different models in capturing historical systemic risk patterns. For instance, "Model A demonstrates a lower rejection rate in the coverage test" rather than "Model A outperforms Model B."

These revisions ensure that the evaluation methodology aligns with the nature of our contribution, which we believe directly addresses the underlying scientific concern the Reviewer has raised.

**3. While high-dimensional problems would genuinely arise with proper out-of-sample evaluation using rolling windows, the authors’ in-sample approach across the full 10+ years of weekly data (that should be daily) with 55 assets makes the “curse of dimensionality” argument questionable. With T ≫ N in their full-sample estimation, multivariate models are feasible.**

***Response to Reviewer#1 Comment 3:*** High-Dimensional Claims and Benchmarking

We sincerely thank the Reviewer for the suggestion to include the multivariate DCC-GARCH model as a like-for-like benchmark. We fully agree that this would be the natural comparator and have invested substantial effort in attempting its implementation. Unfortunately, our experience reveals that DCC-GARCH is computationally intractable at the scale of our empirical application, a finding that is itself informative and that we wish to transparently document.

(1). Standard DCC-GARCH: Computational intractability at N = 55

We attempted to estimate the standard DCC (1,1) model using the MFE Toolbox in MATLAB on our full dataset of 55 financial institutions over 626 weekly observations. The estimation was performed on a workstation with an Intel Core i9-9900KF processor (3.6 GHz). While the model parameters converged within minutes, the routine became computationally intractable when we requested the full sequence of conditional covariance matrices Ht—a necessary output for constructing the CoVaR and CoES forecasts required by our backtesting framework. The estimation did not return Ht within 24 hours of execution time.

To verify that this is a dimensionality issue rather than a coding or hardware problem, we reduced the cross-section from 55 to 10 institutions and re-ran the identical procedure. With N = 10, the full Ht sequence was successfully returned in approximately 23 minutes. The super-linear scaling of computation time with N confirms that we are encountering the well-documented curse of dimensionality in the standard DCC model: at each time step, a 55 × 55 covariance matrix must be constructed and constrained to positive definiteness, and the computational burden grows prohibitively with the cross-sectional dimension.

(2). DCC-NL: A dimension-robust alternative that yields degenerate dynamics

We then explored the DCC-NL (Nonlinear Shrinkage) estimator proposed by Engle, Ledoit, and Wolf (2019), which is specifically designed to overcome the dimensionality limitations of the standard DCC model. Using the authors' publicly available code, we successfully obtained Ht estimates for the full 55-institution panel within a feasible timeframe via the DCCcov command. However, the resulting conditional correlation matrices exhibited virtually zero time-series variation across the entire sample period. That is, while the DCC-NL estimator is dimension-robust, the estimated dynamics were degenerate for our application, yielding essentially static correlation estimates that defeat the purpose of a dynamic benchmark. We therefore conclude that the DCC-NL output is unsuitable for use as a meaningful comparator in our study.

Reference:

Engle, R. F., Ledoit, O., & Wolf, M. (2019). Large dynamic covariance matrices. Journal of Business & Economic Statistics, 37(2), 363–375.

(3). Implications for our study

These findings carry a clear implication for the evaluation framework of our study. The Reviewer correctly identified the need for a like-for-like multivariate benchmark, and our extensive attempts to implement DCC-GARCH and DCC-NL confirm that this is a well-motivated suggestion in principle. However, the practical infeasibility of both approaches at the scale of our empirical application—55 institutions—demonstrates that a viable multivariate GARCH-type benchmark is not readily available for our data structure. This is not a limitation unique to our study; it reflects a broader gap in the literature that our factor copula framework is specifically designed to fill. The factor structure imposes a low-dimensional parameterization on the dependence matrix, circumventing the very curse of dimensionality that renders standard DCC inoperable and DCC-NL uninformative at this scale.

We therefore believe that the static copula benchmarks and time-varying copula benchmarks already included in the paper provide a reasonable and transparent evaluation framework, and that the absence of a DCC-GARCH benchmark does not compromise the integrity of our empirical design. The computational evidence documented here reinforces, rather than weakens, the methodological motivation for our approach. We appreciate the Reviewer for prompting us to investigate this issue rigorously, as the resulting evidence strengthens the case for factor-based dimensionality reduction in high-dimensional systemic risk applications.

**4. Despite claiming DCC-GARCH “performed poorly,” no specifications, estimation results, or diagnostic evidence is provided. The evaluation protocol lacks critical details about parameter updating, window specifications, and model re-estimation frequency.**

***Response to Reviewer#1 Comment 4:*** Methodological Transparency and Replicability.

We sincerely thank the Reviewer for identifying this critical oversight. The Reviewer is entirely correct: our original submission lacked the necessary documentation for the benchmark models—including specifications and estimation details—rendering our results unreplicable and our exclusion claims arbitrary rather than evidence-based. This was a failure on our part, and we apologize for it.

We have taken the following corrective actions. We have prepared and will submit complete replication code for all benchmark models, including the bivariate dynamic copula, bivariate static copula, and all other comparative specifications used in the paper. We appreciate the Reviewer for holding us to this standard, which has materially improved the transparency and credibility of our study.

Reviewer #2

Reviewer #3

**1. The backtesting framework would benefit from a more intuitive explanation. In particular, I suggest adding a short paragraph outlining the intuition behind the cumulative joint violation process and clarifying how it differs in practice from existing ES/CoVaR backtesting approaches. This would make the contribution more accessible to a broader audience.**

***Response to Reviewer#3 Comment 1:*** Intuitive Explanation of the Backtesting Framework

We thank the Reviewer for this constructive suggestion. We fully agree that a more intuitive exposition of the cumulative joint violation process would make our methodological contribution accessible to a broader audience.

In response, we have added a new paragraph at the end of Section 2.3 (line 275) that develops the intuition from first principles:” The logic of the cumulative joint violation process $H_{t}$ is best understood by starting from the standard univariate VaR backtest and extending it along two dimensions. The first dimension is cumulative: moving from VaR to ES backtesting. In a VaR backtest, one simply counts violations—days when the realized loss exceeds the predicted VaR. ES backtesting goes further by asking whether, on violation days, the magnitude of the loss is consistent with the predicted ES. This requires tracking the cumulative sum of scaled violations over time, rather than merely their frequency. The second dimension is joint: moving from VaR to CoVaR backtesting. Instead of asking whether one institution experiences a violation, we ask whether, conditional on institution i being in distress, the loss of institution j exceeds the predicted CoVaR. This defines a joint violation—a co-exceedance of two thresholds across two institutions. Our CoES framework naturally combines these two extensions: we construct a cumulative joint violation process that tracks, over time, whether the co-exceedances between institution pairs are consistent with the predicted CoES, both in frequency and in serial dependence.” We appreciate the Reviewer for prompting this improvement to the manuscript's clarity.

**2. A brief discussion of the robustness of the simulation-based estimation would also be helpful. For instance, the authors could provide a short justification for the choice of 10,000 simulations, or include a qualitative remark on the sensitivity of results to this parameter.**

***Response to Reviewer#3 Comment 2:*** Robustness of Simulation-Based Estimation

We thank the Reviewer for this suggestion. We note that the manuscript already contains a discussion addressing this point. In Section 3.2 (line 363), we provide a convergence diagnostic for our simulation-based estimates. Specifically, we examine the tail dependence coefficient (TDC) and tail correlation (TC) for four randomly selected institution pairs over the final 2,000 simulation runs, and confirm that both measures stabilize well before 10,000 simulations—with fluctuations falling below thresholds of 0.05 and 0.08, respectively. We also note that convergence patterns for other randomly sampled pairs are similar. This analysis justifies the choice of 10,000 simulations and demonstrates that our results are insensitive to further increases in this parameter. We appreciate the Reviewer for prompting us to highlight this existing content.

**3. The economic interpretation could be further strengthened by making the distinction between spillover and contagion effects more explicit in economic terms and by linking the findings more directly to the existing literature.**

***Response to Reviewer#3 Comment 3:*** Economic Interpretation — Spillover vs. Contagion and Literature Linkage

We thank the Reviewer for this insightful suggestion. We agree that a sharper distinction between spillover and contagion, combined with a more direct link to the existing literature, would strengthen the economic interpretation of our findings.

In our framework, this distinction arises naturally from the directional structure of the tail-risk network: out-degree captures risk spillover from an institution to others, while in-degree captures risk contagion that an institution receives from distressed peers. In response to the Reviewer's suggestion, we have added a short passage at the end of Section 4 (line 597) that connects this directional decomposition to the established taxonomy in the systemic risk literature. The added text reads: " This distinction aligns with the systemic risk literature, where spillover (out-degree) refers to shock propagation through fundamental linkages, and contagion (in-degree) denotes vulnerability to tail events transmitted from distressed peers—a phenomenon that intensifies during crises [4,29]. Our finding that institutional factors primarily drive spillover while market factors shape contagion complements existing evidence on the distinct determinants of systemic risk transmission and vulnerability."

**4. The discussion of counterintuitive findings (e.g., leverage and local government debt variables) is appreciated. However, it would be useful to emphasize these interpretations more clearly in the main discussion and to clarify whether these findings are likely to be context-specific or driven by modeling choices.**

***Response to Reviewer#3 Comment 3:*** Discussion of Counterintuitive Findings

We thank the Reviewer for this suggestion. We agree that the counterintuitive regression results warrant a clearer contextualization in the main discussion.

In the first round of revision, we relocated detailed speculative interpretations of these anomalies to the "Future Research" section to keep the main discussion focused on our core findings. In this round, we have added a bridging sentence in Section 4 (line 556) immediately following the presentation of the counterintuitive result. The added sentence reads: "These counterintuitive patterns regarding the liability variable and the dgo variable likely stem from the unique institutional structure of China's financial system, which differs substantially from the mature markets that underpin standard theoretical predictions." This sentence signals to readers that the anomalies are plausibly context-specific, while the "Future Research" section provides the full interpretive discussion. We believe this adjustment addresses the Reviewer's concern without disrupting the manuscript's established structure. We appreciate the Reviewer for prompting this refinement.

**5. Minor improvements:
- Some parts of the methodology remain quite dense; a short guiding paragraph could improve readability.**

**- A compact table summarizing notation could help readers navigate the model more easily.**

**- Figure captions could be slightly expanded to improve standalone readability.**

**- The resolution of figures should be improved.**

***Response to Reviewer#3 Comment 5:*** Minor Improvements

We thank the Reviewer for these constructive suggestions. We have addressed each point as follows:

Methodology readability. The backtesting methodology, particularly the cumulative joint violation process, is indeed the most technically dense portion of the manuscript. In response to the Reviewer's first comment, we have already added an intuitive exposition of the "cumulative" and "joint" dimensions at the end of Section 2.3 (line 275), unpacking these concepts from the standard univariate VaR backtest. We believe this addition also serves the purpose of a guiding overview for the methodological section and substantially improves its accessibility.

Notation table. We appreciate this suggestion. In the first round of revision, we carefully ensured that every newly introduced symbol is defined immediately upon its first appearance in the text. Given that all notation is already explained in close proximity to its point of introduction, we hope the Reviewer finds the current presentation adequate. That said, if the Reviewer or Editor deems a separate notation table necessary, we are happy to add one.

Figure captions. We have expanded some figure captions to include a brief description of what is plotted, the sample period covered, and the key pattern the reader should observe, ensuring that each figure is interpretable on a standalone basis.

Figure captions and resolution. We have regenerated all figures—particularly the network topology trend plots—at 300 DPI resolution and replaced the original lower-resolution versions. The revised figures are now approximately 2 MB each, ensuring clear legibility in both electronic and print formats.

Thank you very much for your attention and time. Look forward to hearing from you.

Yours sincerely.

Feng Shen, Professor

shenfeng@swufe.edu.cn
